# Atomically Precise Metal Nanoclusters for Bioimaging and Therapy: Progress and Perspectives

**DOI:** 10.1021/jacsau.5c01206

**Published:** 2025-11-05

**Authors:** Tingting Xu, Beibei Xiang, Yu Chen, Yitong Wang, Yongbo Song, Rongchao Jin

**Affiliations:** † School of Biomedical Engineering, Research and Engineering Center of Biomedical Materials, 12485 Anhui Medical University, Hefei 230032, P. R. China; ‡ Department of Chemistry, 6612 Carnegie Mellon University, Pittsburgh, Pennsylvania 15213, United States

**Keywords:** Atomically precise metal nanoclusters, physicochemical properties, bioimaging, therapy

## Abstract

Nanomaterials that simultaneously possess bioimaging and therapeutic functions along with excellent biocompatibility have long been pursued for early disease detection as well as precise therapy, which is critical for enhancing the cure rates and quality of life for patients. The recent advent of atomically precise metal nanoclusters (MNCs) is expected to contribute to the realization of this goal. MNCs are of ultrasmall size (<3 nm) and truly monodisperse, comprising a specific number of atoms in the range of several to hundreds of metal atoms, which endows them with efficient renal clearance, thereby ensuring their excellent biocompatibility. Furthermore, MNCs with discrete electronic energy levels can be tailored to exhibit superior photoluminescence in the near-infrared region (e.g., 1000–1700 nm), and MNCs are also characterized by exceptional photostability and large Stokes shifts. All these features make them excellent candidates as bioimaging probes with high detection sensitivity, deep tissue penetration, and high spatiotemporal resolution. Moreover, due to the wide-range optical absorption and enzyme-like catalytic activity, MNCs also provide various effective therapeutic strategies, including photothermal therapy, photodynamic therapy, chemodynamic therapy, and radiotherapy. Importantly, with the ligand-engineering and alloying strategies, MNCs can be used in imaging-guided precise therapy. In this Perspective, we first introduce the biologically related properties of MNCs, then present the applications of MNCs in bioimaging and therapy, and finally discuss some existing challenges and future prospects. We hope that this Perspective will stimulate broader interest to multidisciplinary researchers, and the collective efforts will boost the applications of atomically precise MNCs in biomedicine.

## Graphical abstract

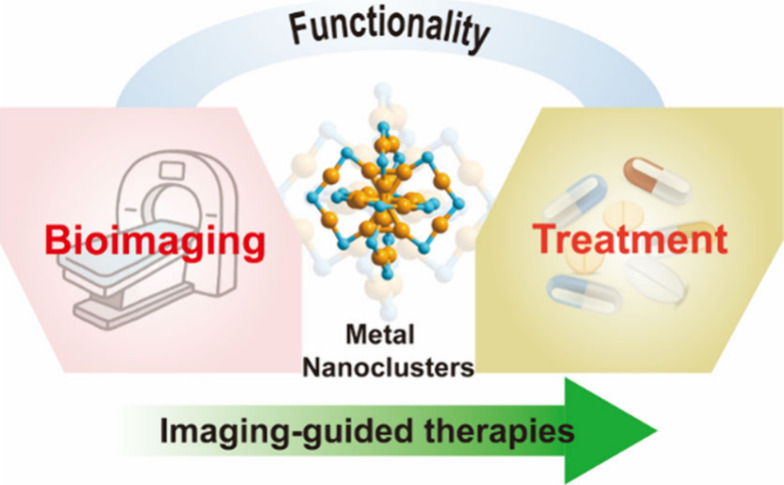

## 1 Introduction

The rapid advancement of nanomaterials (NMs) has effectively addressed critical challenges across multiple areas, particularly in biomedicine.^1–3^ These materials have demonstrated remarkable potential in (i) enhancing imaging and diagnostic technologies,^4–6^ (ii) enabling precision drug delivery systems,^7–9^ (iii) facilitating synergistic disease therapies,^10–12^ and (iv) promoting tissue regeneration.^13–15^ However, a fundamental challenge still persists for nearly all biomedical NMs: ensuring biosafety. Conventional nanoparticles (NPs) often exhibit tendencies toward aggregation, forming large aggregates that resist metabolic clearance, hence posing a major safety concern. Mechanistically, such an accumulation in tissues and organs may trigger adverse effects, ranging from cellular apoptosis to tissue necrosis.^16^ Therefore, optimizing NM biosafety through structural modifications, size control, and surface engineering remains imperative.^17,18^

In recent years, atomically precise metal nanoclusters (MNCs) have emerged as a promising class of materials for nanomedicines, such as disease diagnosis and therapy.^19–21^ Their ultrasmall size (<3 nm), which lies below the renal filtration threshold (∼5.5 nm), ensures efficient renal clearance, while their compact dimensions confer favorable pharmacokinetic profiles. Both *in vitro* and *in vivo* studies have further validated their exceptional biosafety.^22–24^

Unlike larger-sized NPs (tens of nanometers) which possess continuous electronic energy bands and thus plasmon properties, MNCs instead exhibit discrete levels due to quantum confinement effects when their metal core approaches the Fermi wavelength of electrons (∼0.5 nm).^25–27^ This quantum-scale transition converts continuous electronic bands into discrete energy levels, endowing MNCs with molecule-like behaviors, including distinct highest occupied molecular orbital (HOMO)–lowest unoccupied molecular orbital (LUMO) transitions, tunable optical absorption, magnetism and luminescence.^28–30^ Structurally, MNCs comprise a well-defined metal core stabilized by organic ligands, ensuring true monodispersity and high colloidal stability—critical features that effectively suppress aggregation.^30,31^ Taken together, all the attributes—superior biocompatibility, exceptional physicochemical properties, and robust stability—establish MNCs as pivotal candidates for advanced biomedical applications.^32–35^

In this Perspective, we focus on atomically precise MNCs, offering a comprehensive overview of their biologically relevant properties and applications in bioimaging and therapeutic modalities (Scheme 1). Some of the atomically precise MNCs, especially those frequently applied in biomedicine such as Au_25_(SR)_18_, are presented in Figure 1, with their properties and applications presented in later sections. Following the sections of physicochemical properties (section 2), bioimaging (section 3), disease treatment (section 4) and imaging-guided therapy (section 5), we further outline the current challenges associated with the biomedical use of atomically precise MNCs and discuss potential directions for future research. It is hoped that this review will boost the ongoing efforts in the exploration of novel applications of MNCs in the biomedical field.

**Scheme 1:**
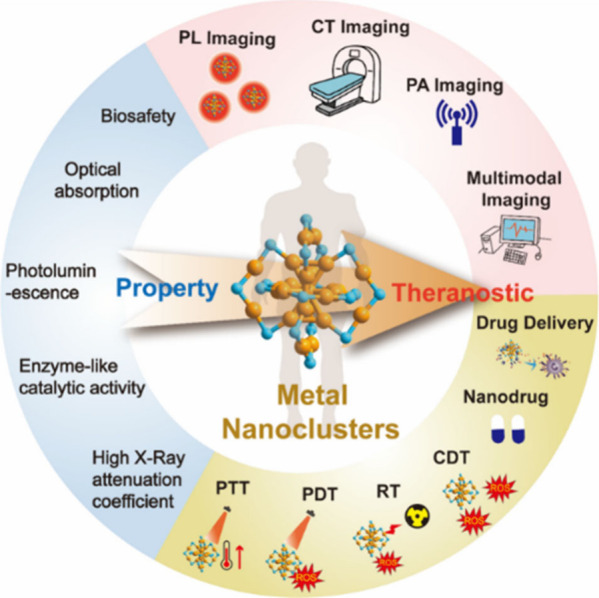
Schematic Illustration of MNCs with Biologically Relevant Characteristics and Biomedicine Applications

**Figure 1:**
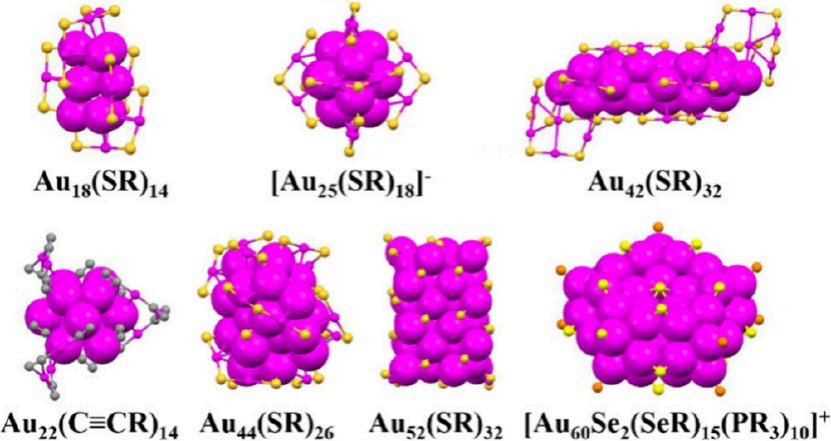
Some representative MNCs and their atomic structures determined by X-ray crystallography. Color code: magenta = Au, yellow = S (or Se), gray = C, orange = P, all −R groups are omitted. Redrawn from the cif files.

## 2 Physicochemical Properties of MNCs

MNCs have emerged as a new class of molecule-like functional NMs, displaying a variety of intriguing physicochemical properties, such as multiple absorption bands,^36–38^ photoluminescence (PL),^39–41^ catalytic activity,^42–44^ chirality,^45–47^ and magnetism,^48,49^ which make them attractive for applications in multiple fields.^50–53^ Furthermore, the atomically precise structures of MNCs provide a unique opportunity to investigate the correlation between their structures and properties.^28,30,54,55^ By controlling the structures (geometric or electronic), atomic composition, and capping ligands at the atomic/molecular level, the physicochemical properties of MNCs can be tailored to achieve desired functionalities.^56–58^ MNCs with tunable properties have been widely recognized as promising theranostic probes for clinical translation. In this section, we discuss the correlation between the fundamental properties and practical applications of MNCs by highlighting their biologically relevant characteristics. These fundamental properties constitute the basis for their biomedical applications (Scheme 2).

**Scheme 2:**
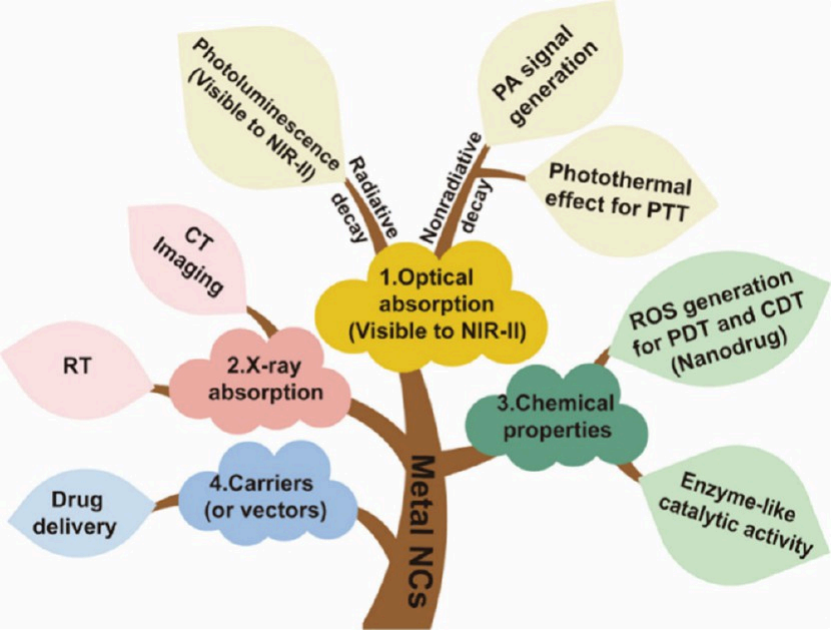
Fundamental Properties of MNCs and Applications in Biomedicine

### 2.1 Biocompatibility and Biosafety

Having a good biosafety is a fundamental prerequisite for the application of NMs in the biomedical field, requiring low cytotoxicity, minimal metabolic toxicity, and negligible harmful effects on organs.^59–61^ First of all, both the metal core and ligand shell of MNCs can influence the cytotoxicity. Therefore, biocompatibility can be improved through the use of hydrophilic and biocompatible ligands, such as glutathione (GSH), cysteine (Cys), bovine serum albumin (BSA), captopril (Capt), and 3-mercaptopropionic acid (MPA).^62–65^ Second, renal clearance constitutes the primary excretion pathway for ultrasmall metal NPs (hydrodynamic diameter <6 nm).^22,66^ Zheng and colleagues reported that the glomerular filtration barrier exhibits atomic-level precise band-pass filtering behavior, which can significantly reduce the clearance rate of small gold nanoclusters (Au NCs) in the kidneys (Figure 2A).^23^ Compared with Au_25_ (∼1.0 nm), Au NCs with the same surface ligands but smaller sizes (Au_18_, Au_15_, and Au_10–11_) show a marked decrease in early renal clearance efficiency despite only a few atoms less (Figure 2B). This phenomenon can be attributed to the greater retention of smaller Au NCs by the glomerular glycocalyx compared with larger ones (Figure 2C). These findings suggest that smaller NPs are not necessarily more efficiently cleared via the renal pathway. Additionally, several other factors, including surface charge, stability, and shape, significantly influence renal clearance.^67^

**Figure 2:**
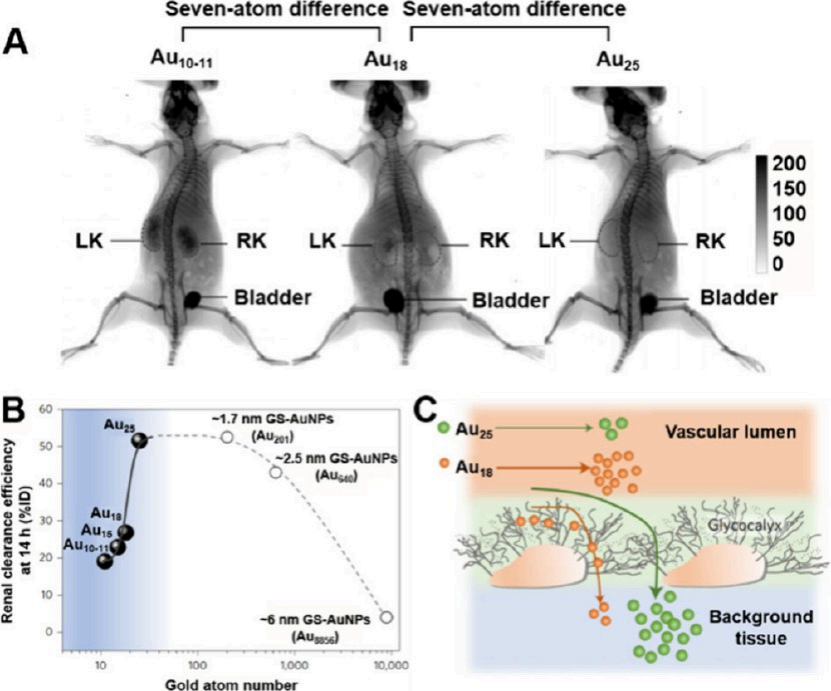
(A) Whole-body X-ray images of mice after being intravenously injected with Au_10–11_, Au_18_ or Au_25_ NCs at 40 min post injection. (B) Renal clearance efficiencies of different NCs at 24 h post injection versus the number of gold atoms in the NC. (C) Schematic diagram of Au_18_ and Au_25_ crossing the blood vessel walls. Reproduced with permission from ref 23, Copyright 2017, Springer Nature.

Large-sized NMs with unfavorable metabolic profiles are prone to accumulate in the liver, potentially leading to chronic inflammation and fibrosis, which may ultimately impair overall health.^68,69^ Therefore, NMs intended for *in vivo* applications must exhibit minimal or no adverse effects on vital organs, including the heart, liver, spleen, lung, and kidney. Ensuring this biosafety profile is critical to preventing disruption or impairment of their normal physiological functions. In addition to the large-size-induced bioaccumulation and toxicity of some of the conventional NMs, the impurities such as residual gold salt (AuCl_4_^–^) and the protecting surfactants are highly toxic to cells but are not easy to be completely removed. In contrast, atomically precise gold nanoclusters are of molecular purity and do not pose the above concerns.^23,30^ In addition, such MNCs do not have surface defects as in conventional nanoparticles; thus, MNCs can uniformly interact with targets, and the concerns of defect-induced uncontrollable interactions with biological issues are eliminated. Therefore, MNCs are much more biocompatible and safer than conventional nanomaterials for biomedical applications.

Zhang et al. evaluated the hematological, tissue, and neurological impacts of Au_25_(MPA)_18_ and Au_24_Zn_1_(MPA)_18_ following exposure to high doses (300 and 500 mg kg^–1^).^70^ The results showed that biochemical and hematological parameters remained within normal reference ranges even after administration of MNCs at an extremely high dose of 500 mg kg^–1^. Moreover, no histopathological changes were detected, and immunofluorescence staining revealed no significant lesions in the major organs. Hence, to achieve favorable biosafety profiles, it is essential to carefully select both the metal core and surface ligands. The metal core should be chosen based on its intrinsic biocompatibility, while surface ligands should be selected according to their capacity for mild and nondisruptive interactions with biological molecules.

### 2.2 Optical Absorption

Optical absorption is the prerequisite to the generation of many signals for biomedical utilization, such as the photoluminescence via radiative decay of electrons in excited states, photoacoustic, and photothermal signals via nonradiative decay (Scheme 2).

It is widely recognized that spherical gold NPs in the 5–20 nm size range exhibit a prominent absorption peak at ∼520 nm due to the surface plasmon resonance (SPR) phenomenon.^71^ However, as the particle size decreases, the SPR absorption characteristics gradually fades out.^30^ When the size is reduced to below 3 nm—approaching the Fermi wavelength of electrons—the motion of electrons becomes restricted.^72^ This confinement results in the transformation of previously continuous energy bands into discrete electronic energy levels. Consequently, ultrasmall MNCs exhibit discrete molecular-like absorption peaks arising from ground-state electronic transitions.^73,74^

In 2008, Zhu et al. reported the structure of [Au_25_(PET)_18_]^−^ (PET = 2-phenylethanethiolate) and Aikens et al. employed time-dependent density functional theory (TD-DFT) calculations to analyze the electronic properties of [Au_25_(PET)_18_]^−^.^75^ As shown in Figure 3A, the TD-DFT results show that the electronic orbitals are significantly quantized, and the long-wavelength absorption peak of [Au_25_(PET)_18_]^−^ originates from the electronic transition between the HOMO and the LUMO. Specifically, the absorption peaks observed at 2.91, 2.63, and 1.52 eV correspond to d→sp, sp→sp/d→sp, and sp→sp electronic transitions, respectively—features that are fundamentally different from the absorption characteristics of larger Au NPs (Figure 3B).

**Figure 3:**
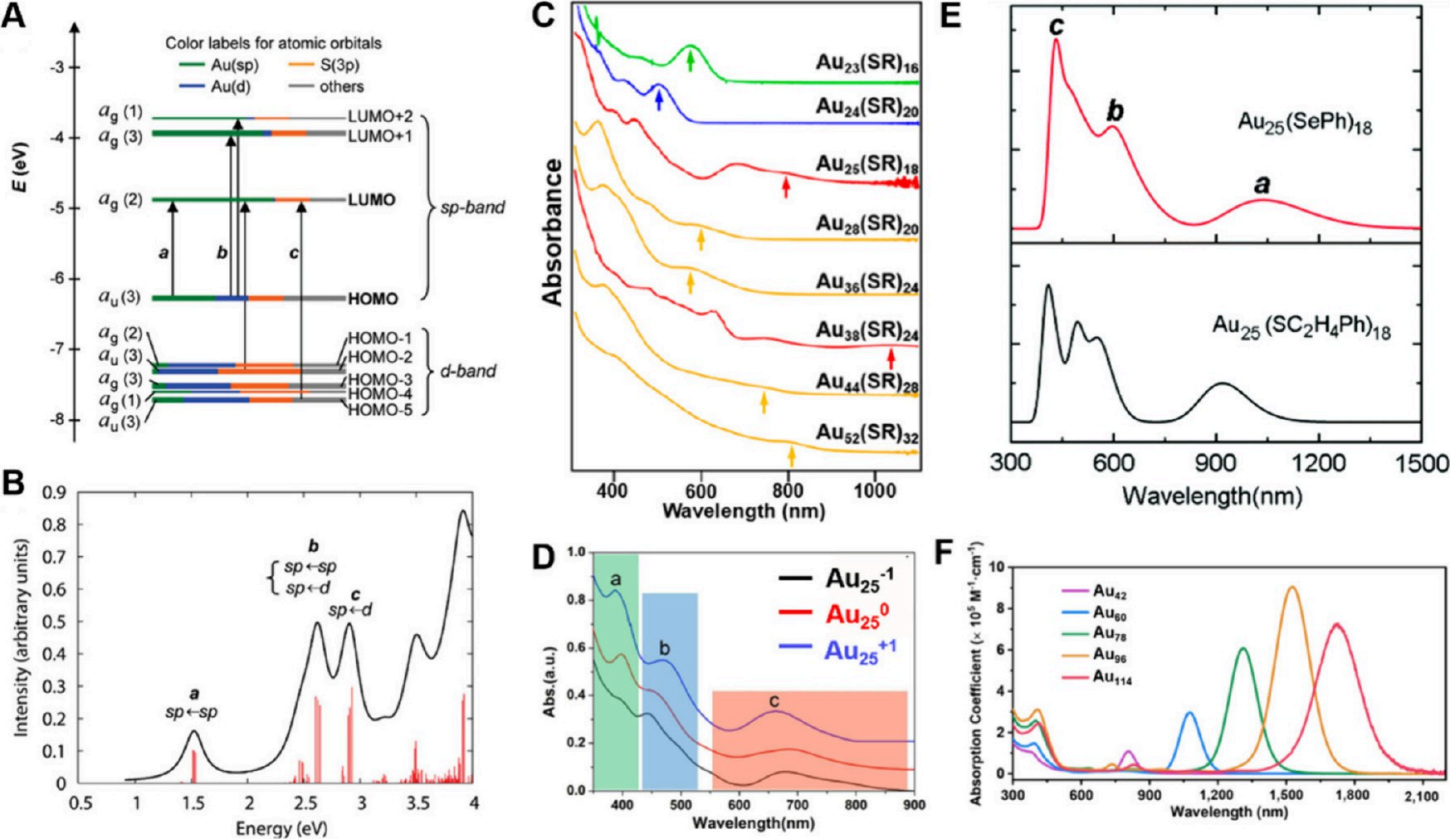
(A,B) Kohn–Sham orbital level diagram and peak assignments of the absorption spectrum of [Au_25_(S-PhC_2_H_4_)_18_]^−^ NCs. Reproduced with permission from ref 75, Copyright 2008, American Chemical Society. (C) UV–vis absorption spectra of a series of Au NCs. Reproduced with permission from ref 76, Copyright 2019, American Chemical Society. (D) Absorption spectra of Au_25_(S-PhC_2_H_4_)_18_ NCs with −1, 0, and +1 charge states. Reproduced with permission from ref 77, Copyright 2018, Royal Society of Chemistry. (E) Comparison of theoretical optical curves of Au_25_(SePh)_18_ and Au_25_(SC_2_H_4_Ph)_18_ NCs. Reproduced with permission from ref 78, Copyright 2014, Royal Society of Chemistry. (F) Absorption spectra of Au_42_, Au_60_, Au_78_, Au_96_, and Au_114_, respectively. Reproduced with permission from ref 79, Copyright 2024, National Academy of Sciences U.S.A.

The optical absorption properties of MNCs are strongly influenced by factors such as the size, charge, and type of capping ligands. Jin et al. conducted a comprehensive analysis and comparative study of the optical absorption characteristics of thiolate-protected Au NCs containing different numbers of Au atoms, aiming to explore the systematic variations in their optical behavior.^76^ The findings indicated that for small-sized NCs (ca. <50 Au atoms), the optical properties are primarily determined by the detailed structural configuration of NCs rather than their size (Figure 3C). For medium-sized NCs (approximately 50–100 Au atoms), both size and structure contribute to the observed optical behavior. In contrast, in the case of larger Au NCs (ca. >100 Au atoms), the optical properties are predominantly governed by the size, while the influence of structural factors becomes considerably weaker. It was demonstrated that the optical absorption of Au_25_(PET)_18_ NCs becomes different when in different charge states,^77^ in which the HOMO – LUMO transition shows a progressive enhancement as the charge state shifting from – 1 to 0 and then to +1 (Figure 3D). Additionally, Au_25_ NCs capped by different ligands (e.g., -SePh) were designed to explore the effects of ligands on the optical prpperties.^78^ TD-DFT calculations demonstrated that changing the ligands from thiolate to selenolate not only can induce a red shift in the absorption peak (Figure 3E), but also can decrease the HOMO and LUMO energy levels (become more negative), thereby enhancing the overall stability of the Au_25_ NCs.

It is widely recognized that the absorption spectra of most MNCs display multi-step-like characteristics, where the absorbance gradually decreases with increasing wavelength, especially for the near-infrared region (NIR). Notably, the Jin group has successfully synthesized a series of gold NCs through precise regulation of their one-dimensional growth mode, including Au_42_(PET)_32_, Au_60_(PET)_44_, Au_78_(PET)_56_, Au_96_(PET)_68_, and Au_114_(PET)_80_.^79^ These Au NCs exhibit strong absorption in both NIR-I and NIR-II. As can be seen from Figure 3F, all the NCs exhibit an absorption peak at ∼ 400 nm and another peak from 806 to 1727 nm depending on the rod’s length. The phenomenon of intense NIR absorption (molar absorption coefficients of 10^5–6^ M^–1^ cm^–1^) is exceptionally rare in NCs.

In addition, intramolecular assembly—in which the basic building blocks are integrated by sharing a vertex, an edge, or a face — is widely recognized as an effective strategy for tuning the optical properties of MNCs across the visible to NIR region.^80^ Taking the classic icosahedral Au_13_ building block as an example, the monomeric Au_13_ nanocluster shows two bands at 360 and 490 nm in its UV–vis optical spectrum.^81^ As the icosahedral Au_13_ units are linearly assembled by vertex sharing in the bi-icosahedral Au_25_ and tri-icosahedral Au_37_ NCs, the energy gap shifts from ∼490 to 670 nm (for bi-icosahedral Au_25_) to 1230 nm (for tri-icosahedral Au_37_).^82,83^ This can be attributed to the electronic interaction between the neighboring Au_13_ units, where the high symmetry is disrupted, causing the atomic orbitals aligned along the rod-like molecule’s long axis (the *z* axis) to have lower energy, while those along the x and *y* axes have higher energy.^84,85^ Similarly, a smaller optical energy gap (∼835 nm) was also observed in the cyclic penta-icosahedral Au_60_ NC.^84^ It is important to note that, regardless of the assembly manner, the two absorption peaks at ∼430 and ∼490 nm can be assigned to the electronic transitions within individual Au_13_ units since the spherically symmetric *s* orbitals are unaffected by symmetry breaking and their orbital energies remain unchanged.^85^

Overall, the visible to NIR absorption properties of the MNCs have very promising applications in fields such as photothermal therapy (PTT), photodynamic therapy (PDT), and photoacoustic (PA) imaging, which will be further discussed in the following sections.

### 2.3 Photoluminescence

It has been shown that PL is a key characteristic of MNCs, offering a novel platform for creating luminescent probes for practical uses such as sensing and biomedical applications, thanks to their excellent photostability and large Stokes shift.^86–88^ Recently, with improvements in precise structural control and theoretical modeling, more luminescent MNCs have been synthesized and various methods to boost their luminescence have been developed.

First, ligand engineering plays a crucial role in adjusting the PL performance of MNCs by effectively modulating the charge transfer between ligands and metal core (i.e., LMCT) through Au–S bonds or the donation of delocalized charge densities from ligands to metal core using the ligands with electron-donating groups (EDGs) or electron-withdrawing groups (EWGs).^88–90^ For example, Wu et al. previously studied how ligands affect the PL of [Au_25_(SR)_18_]^−^ NCs, in which three kinds of [Au_25_(SR)_18_]^−^ NCs (R = SC_2_H_4_Ph, C_12_H_25_ and C_6_H_13_) were synthesized.^91^ They found that the PL intensity of these species follows the order of [Au_25_(SC_6_H_13_)_18_]^−^ < [Au_25_(SC_12_H_25_)_18_]^−^ < [Au_25_(SC_2_H_4_Ph)_18_]^−^, which is parallel to the order of charge-donating capability of the ligands (C_6_H_13_ < C_12_H_25_ < PhCH_2_CH_2_). Kang et al. investigated the PL of Au_2_Cu_6_ NCs (Figure 4A), which was tailored by modifying the phosphine ligands with different groups (P­(Ph-F)_3_, PPh_3_, and P­(Ph-OMe)_3_).^92^ The PL quantum yield (QY) was greatly improved from 5.7% (P­(Ph-F)_3_) to 12.2% (PPh_3_) and further to 17.7% (P­(Ph-OMe)_3_).^92^

**Figure 4:**
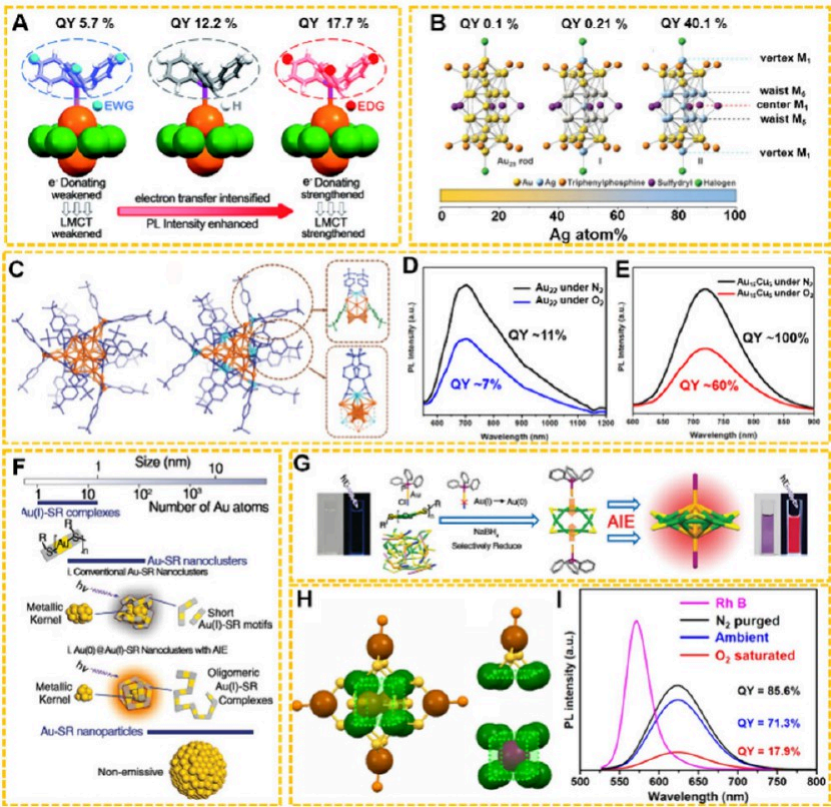
(A) Illustrations of Au_2_Cu_6_ NCs PL intensity enhancement are induced by the phosphine ligands (orange = Au; green = Cu; yellow = S; purple = P; cyan = F; gray = C and light gray = H). Reproduced with permission from ref 92, Copyright 2017, The Royal Society of Chemistry. (B) X-ray structures of controllable Au–Ag composition in Ag_*x*_Au_25‑*x*_(PPh_3_)_10_(SR)_5_Cl_2_ NCs, and the corresponding PL QY. Reproduced with permission from ref 96, Copyright 2014, Wiley-VCH. (C) Topological structures and the corresponding motifs of Au_22_ and Au_16_Cu_6_ (orange = Au; light blue = Cu; blue/deep blue or green = C). Comparison of the emission spectra of (D) Au_22_ and (E) Au_16_Cu_6_ under different condition. Reproduced with permission from ref 97, Copyright 2024, American Association for the Advancement of Science. (F) Structures of (i) conventional Au nanoclusters with short Au­(I)–SR motifs and (ii) luminescent Au nanoclusters with long Au­(I)-SR motifs possessing AIE behavior. Reproduced with permission from ref 98, Copyright 2012, American Chemical Society. (G) Illustration of the Au_0_-induced aggregation of CuSR_1_ and the aggregation-induced PL intensity enhancement (gold = Au, blue = Cl, green = Cu, violet = P, yellow = S). Reproduced with permission from ref 101, Copyright 2016, Wiley-VCH. (H) The Cu_14_(SR)_12_(BCPP)_6_ cage, the Cu­(SR)_2_(BCPP) mount motif, and the body-centered cubic Au@Cu_8_ (magenta = Au; brown = octahedral Cu; green = cubic Cu; yellow = S; orange = P). (I) Comparison of the emission spectra of a CH_2_Cl_2_ solution of Au@Cu_14_ under different conditions. Reproduced with permission from ref 102, Copyright 2021, American Association for the Advancement of Science.

Second, alloying (or heteroatom doping) is an effective approach for adjusting the PL properties of MNCs.^93–95^ Compared with the monometallic NCs, alloying can not only effectively regulate the electronic structure of MNCs, especially the HOMO–LUMO gap, but also control the charge/electron transfer between metal atoms due to the orbital hybridization occurring between different kinds of metal atoms. In 2014, Wang et al. successfully incorporated Ag atoms into rod-like M_25_ NCs by different methods (Figure 4B): (1) reacting Au_11_ NCs with Ag­(I)-SR complex to form the [Ag_*x*_Au_25‑*x*_(PPh_3_)_10_(SC_2_H_4_Ph)_5_Cl_2_]^2+^ (*x* ≤ 13) NCs; whereas (2) reaction of Ag­(I)-SR with the PPh_3_-capped Au nanoparticles produced [Ag_*x*_Au_25‑*x*_(PPh_3_)_10_(SC_2_H_4_Ph)_5_Cl_2_]^2+^ (*x* ≤ 12) NCs. Compared to the homogold­[Au_25_(PPh_3_)_10_(SC_2_H_4_Ph)_5_Cl_2_]^2+^, the PL intensity of Ag-doped M_25_ NCs was significantly improved.^96^ Notably, the Ag_*x*_Au_25‑x_ (*x* ≤ 13) NCs showed a significantly enhanced PLQY (40.1%). Recently, Wang et al. synthesized Au_22_ and its isostructural Au_16_Cu_6_ NCs (Figure 4C), and found that Cu doping significantly suppressed the nonradiative decay, with a reduction of ∼ 60-fold, while simultaneously enhancing the intersystem crossing rate (∼300-fold), and the PL intensity was significantly enhanced (Figure 4D,E).^97^ The Au_16_Cu_6_ NCs exhibited a PLQY exceeding 99% in a degassed solution at room temperature, and maintained a high QY of up to 61% under oxygen-saturated conditions (Figure 4E).

Third, utilizing the distinctive core–shell structure of the MNCs, the concept of aggregation-induced emission (AIE) is frequently applied to design and synthesize MNCs with strong PL,^98–100^ in which the nonradiative decay induced by vibrations or rotations is minimized. In 2012, Xie and co-workers suggested that the AIE approach could be applied to create AIE-type Au(0)@Au­(I)-thiolate NCs,^98^ which achieved strong emission through the controlled aggregation of Au­(I)-SG complexes on the Au(0) core surface (Figure 4F). Inspired by this, some monodisperse MNCs with strong emission have been developed,^101–103^ in which the interactions within the molecule (e.g., aurophilic interactions, chelating coordination between metals and ligands) endow the MNCs with increased rigidity. Zhu and co-workers described a Au_2_Cu_6_ NC exhibiting a QY of 11.7%, where the Cu_6_(SR)_6_ complex was stabilized by two Au(0) atoms (Figure 4G).^101^ Subsequently, they synthesized a AuCu_14_ NC, in which a large Cu_14_(SR)_14_(PR_3_)_6_ complex aggregates on a Au(0) atom, achieving a high QY of 71.3% in nondegassed solution at room temperature (Figure 4H–I).^102^ It is worth mentioning that some MNCs have been found to exhibit strong luminescence in the aggregated or crystalline state, such as Cu_14_ and Au_4_Ag_13_,^104,105^ in which it is the intermolecular forces reduce the nonradiative decay caused by vibrations or rotations.

Compared to bioimaging in the visible light range, a recent trend is to shift to the NIR region, particularly in the NIR-II window (1000–1700 nm) owing to the superior tissue-penetration capability of NIR-II light and reduced autofluorescence/scattering in this region.^106^ Consequently, the development of luminescent NCs with NIR-II emission is of substantial practical importance.^107,108^ Recently, Jin et al. synthesized and systematically characterized four Au_52_(SR)_32_ NCs featuring aromatic thiol ligands (-SR) with different bulkiness at the para-position.^109^ The emission peaks of all four Au_52_(SR)_32_ were observed within the range of 900 to 940 nm. The PLQY exhibited a notable enhancement from 3.8% to 18.3% with a decrease in the ligand’s para-bulkiness (Figure 5A). Song et al. employed the Au_60_ NC as a structural template and incorporated Cu atoms into the metal core, resulting in well-defined alloyed Cu_*x*_Au_61‑*x*_ NCs with dual emission in the NIR-II region (Figure 5B).^110^ The introduction of Cu atoms promotes a more compact structural arrangement (Figure 5C), thereby enhancing spin–orbit coupling and significantly improving the NIR-II luminescence. Sun and his co-workers chose CZ-PrAH as ligands to construct a Au_20_ NC,^111^ which displays NIR dual-emission (820 and 940 nm) with PLQY of 6.26% (Figure 5D,E). Detailed spectroscopic investigation and DFT calculations revealed that both emission peaks originate from the charge transfer from triplet excited state to the ground state, which is rare.^111^ Taken together, the illustrated examples suggest that NIR emission can also be effectively achieved through deliberate modifications of the existing NCs, such as heteroatom doping, ligand engineering, and surface functionalization.

**Figure 5:**
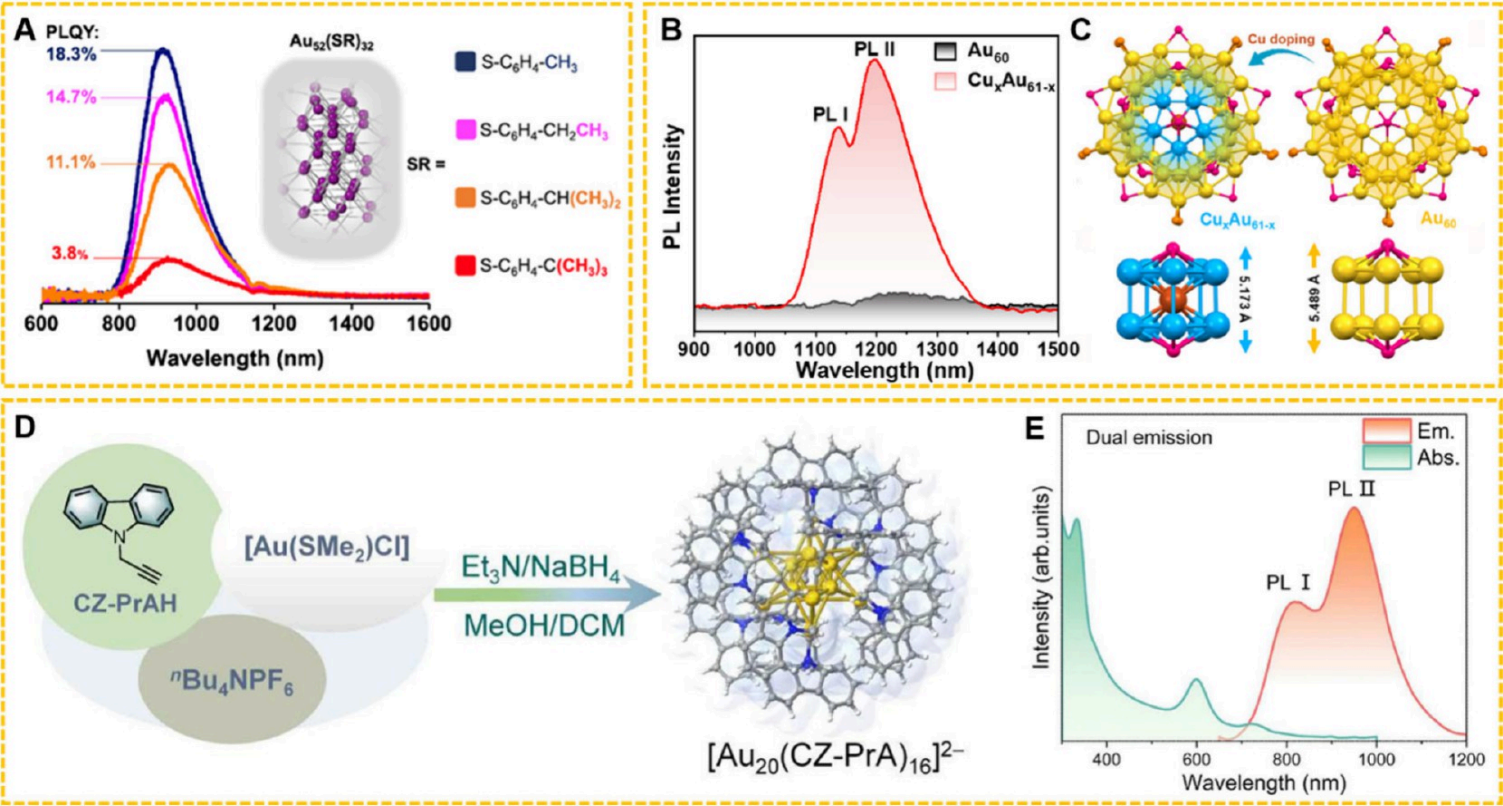
(A) PL and QY of Au_52_(SR)_32_ with different ligands at room temperature in nondegassed CH_2_Cl_2_. Reproduced with permission from ref 109, Copyright 2023, American Chemical Society. (B) NIR-II emission spectra of Cu_*x*_Au_61‑*x*_ compared to Au_60_. (C) Crystal structures and the corresponding metal cores of Cu_*x*_Au_61‑*x*_ and Au_60_ (yellow = Au; blue = Au/Cu; brown = Cu; magenta = Se; orange = P). Reproduced with permission from ref 110, Copyright 2025, Wiley-VCH. (D) Synthetic route for Au_20_ (yellow = Au; blue = N; gray = C; white = H). (E) Absorption and emission spectra of Au_20_ in 2-Me-THF. Reproduced with permission from ref 111, Copyright 2023, American Association for the Advancement of Science.

Overall, MNCs possess several notable advantages in the PL: 1) tunable luminescence spanning the ultraviolet (UV) to the NIR-II region; 2) large Stokes shift, which effectively minimizes interference from excitation light; and 3) excellent photostability, ensuring sustained and reliable luminescence over a long time. These luminescence characteristics make MNCs highly suitable for applications in bioimaging.

### 2.4 Enzyme-like Catalytic Activity

The concept of artificial enzymes was first proposed in the 1970s and has since garnered widespread attention.^112^ With continuous nanotechnology innovations, research on nanozymes began in 2007 when Yan et al. reported the peroxidase-like (POD) activity of Fe_3_O_4_ NPs.^113^ Since then, much research has been conducted on NMs that mimic the structure and catalysis of various enzymes. Compare to natural enzymes, nanozyme have many advantages, such as the tunable catalytic activity, excellent stability, and high selectivity.^114,115^

MNCs generally display active electron (or energy) transfer properties, as well as diverse and tunable metal compositions, leading to favorable catalytic properties.^116^ Therefore, it can be reasonably inferred that MNCs have the potential to exhibit enzyme-like catalytic activities in biomedical applications. Until now, MNCs have exhibited various enzyme-like catalytic activities, including POD-like activity, oxidase (OXD)-like activity, superoxide dismutase (SOD)-like activity, and catalase (CAT)-like activity, etc.

Wu and his colleagues have reported the catalytic activity of well-defined Au_40_(S-Adm)_22_ (S-Adm = adamantanethiolate) NCs as enzyme-like catalysts, broadening the application of NCs in the biocatalysis field.^117^ They bound Au_40_(S-Adm)_22_ with γ-cyclodextrin-metal–organic framework (γ-CD-MOF), resulting in a catalyst that exhibited excellent water solubility and horseradish peroxidase (HRP, i.e., POD)-like catalytic activity (Figure 6A). Afterward, the same group synthesized atomically precise [Au_14_(Dppp)_5_I_4_]^2+^ (Dppp = 1,3-bis­(diphenylphosphino) propane), which demonstrates OXD-like activity by catalyzing the conversion of oxygen (O_2_) into superoxide anion (O_2_^•–^) under visible light irradiation, inducing a blue color change in 3,3′,5,5′-tetramethylbenzidine (TMB) both in solution and in solid state.^118^ The introduction of acetylcholinesterase (AChE) into the blue solution (Au_14_ + TMB) inhibits its OXD-like activity, resulting in the fading of the blue color. However, when organophosphorus pesticides (OPs) are added to the mixture, the activity of the OXD-like is recovered, which leads to the reappearance of the blue coloration (Figure 6B). Zhang et al. successfully synthesized Au_24_Cu_1_ and Au_24_Cd_1_ by doping Cu and Cd into Au_25_ NCs, which exhibited remarkably high antioxidant activity.^119^ Specifically, their antioxidant activities were measured to be 137 and 160 times higher than those of natural trolox, respectively. The Au_25_, Au_24_Cu_1_, and Au_24_Cd_1_ clusterzymes demonstrated notable selectivity in GSH peroxidase-like (GPx-like), CAT-like, and SOD-like activities.^119^ As shown in Figure 6C, Au_25_ shows the strongest GPx-like activity. In contrast, Au_24_Cu_1_, which contains a single Cu atom, exhibits a CAT-like activity. The incorporation of a Cd atom further enhances the selectivity for SOD-like activity. These examples illustrate that MNCs are able to exhibit diverse enzymatic activities and have the potential to serve as alternatives to natural enzymes in biological systems.

**Figure 6:**
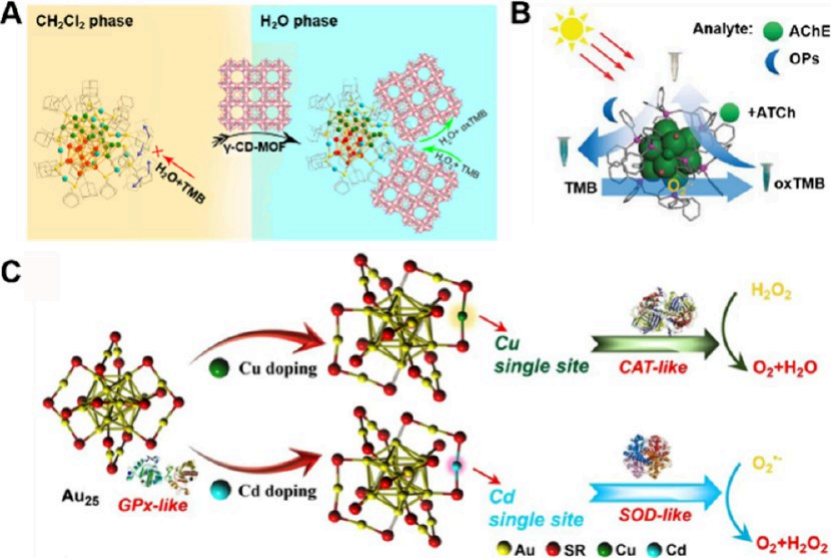
(A) Au_40_(S-Adm)_22_ NCs loaded onto γ-CD-MOF exhibit catalytic activity similar to HRP in aqueous solution. Reproduced with permission from ref 117, Copyright 2020, American Chemical Society. (B) Catalytic process and possible detection mechanism of [Au_14_(Dppp)_5_I_4_]^2+^. Reproduced with permission from ref 118, Copyright 2023, Wiley-VCH. (C) Schematic illustration of the catalytic selectivity of the different clusterzyme systems. Reproduced with permission from ref 119, Copyright 2021, Springer Nature.

### 2.5 High X-ray Attenuation Coefficient

The metal cores within MNCs are typically composed of elements such as Au, Ag, Cu, and Pt. Among these, NCs containing high atomic number (high-Z) elements like Au (Z = 79) and Pt (Z = 78) exhibit strong X-ray attenuation coefficients, high electron densities, and efficient energy deposition characteristics.^120,121^ As is well-known, high-Z materials are capable of efficiently absorbing X- or γ rays, which enables their use as computed tomography (CT) contrast agents in CT imaging for tumor localization, as well as enhancing radiotherapy (RT) sensitization to further eradicate tumor cells.^122^ MNCs with modifiable ligands can be further engineered to specifically target lesion sites, thereby improving the signal-to-noise ratio of the imaging process and minimizing radiation exposure of surrounding healthy tissues.

Owing to these excellent properties discussed in sections of [Sec sec2.1]-[Sec sec2.5], MNCs have garnered significant attention for their promising applications in biomedicine. In recent years, a growing number of studies have investigated the application of MNCs in biological imaging, as well as in therapeutics. Encouraging preliminary results have been obtained in both *in vitro* and *in vivo* experiments. These advancements offer valuable insights into the future development of integrated nanomedicine platforms based on MNCs.

## 3 Bioimaging

Bioimaging plays an important role as an indicator in clinical practice.^123^ For example, bioimaging techniques employed in early disease detection can provide invaluable time for subsequent therapeutic interventions, particularly for rapidly progressing diseases such as highly metastatic malignant tumors.^124^ If cancer can be accurately and promptly detected at an early stage, it will greatly improve patient prognosis and increase the five-year survival rate.^125^ Therefore, compared to formulating an appropriate treatment option, strengthening the diagnosis of cancer is equally crucial for improving the success rate of treatment. In addition to disease diagnosis, visualizing agents can also indicate the distribution and metabolism of drugs in the body through bioimaging, implement monitoring of their dynamic biological processes, and provide a convenient way to study the toxicity of drugs *in vivo*.^34,126,127^ MNCs have demonstrated significant potential in bioimaging, owing to their tunable structures and properties. In this section, we systematically summarize the diverse imaging capabilities of NCs and investigate the underlying mechanisms of their imaging properties.

### 3.1 PL Imaging

As a highly influential optical detection technology in disease diagnosis, PL imaging has been extensively studied.^128^ MNCs with exceptional PL properties have been utilized as effective probes for imaging at subcellular organelle, cells, tissues, and organs.^35^ Compared with other imaging techniques, PL imaging offers the advantages of high sensitivity, high resolution, noninvasiveness and real-time dynamic monitoring.^129^ Furthermore, different from traditional inorganic luminescent materials and organic dyes, MNCs possess characteristic luminescent advantages, including a large Stokes shift, excellent photostability, resistance to bleaching or phototoxicity, and surface facile functionalization, which make them a promising new class of luminescent probe.

Early developed luminescent MNCs primarily emit in the visible light region, leading to numerous reported applications in visible imaging. Gao and his coworks synthesized Au_5_Peptide_3_ and Au_22_Peptide_10_, which were well taken up by human nasopharyngeal cancer cells (CNE1 cells).^130^ To further investigate the subcellular localization of the Au NCs, confocal microscopy analysis revealed that Au_5_Peptide_3_ was predominantly localized in the lysosomes (Figure 7A), with a minor fraction detectable in the mitochondria; whereas Au_22_Peptide_10_ remained largely confined to the lysosomes and had hardly mitochondrial uptake.

**Figure 7:**
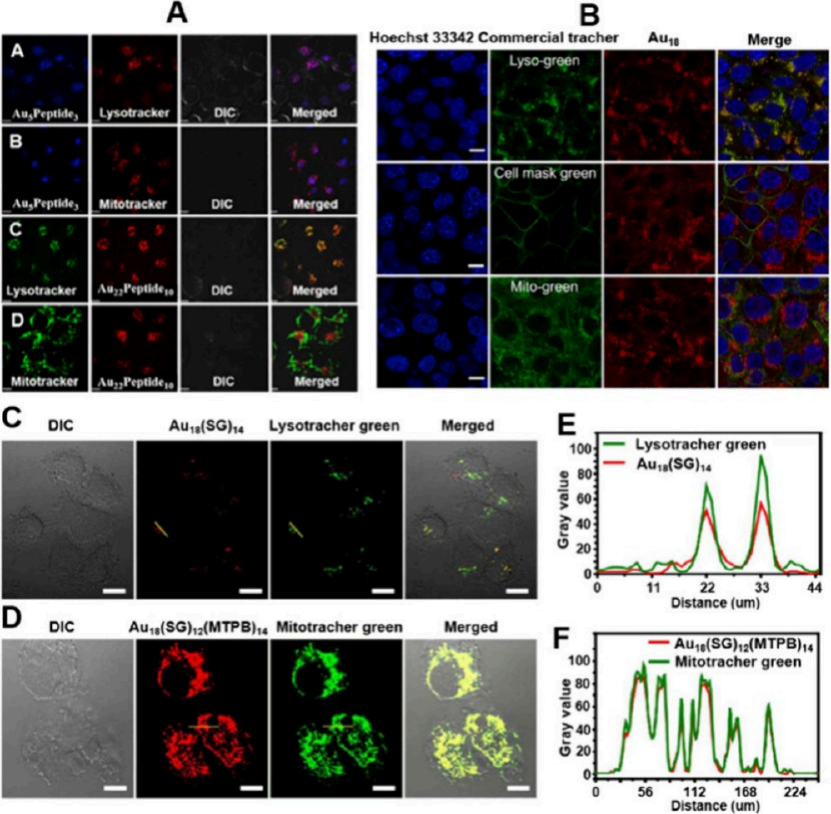
(A) Confocal microscopy images of CNE1 cells exposed to Au_5_Peptide_3_ and Au_22_Peptide_10_ for 24 h at 80 μM Au and then 50 nM lysotracker (panel A, images A and C) or 50 nM Mitotracker (panel A, images B and D) staining cells for 20 min, respectively. Reproduced with permission from ref 130, Copyright 2018, American Chemical Society. (B) Structured illumination microscopy images of 4T1 cells stained with commercial organelle-specific tracker (Hoechst 33342, Lyso-green, Cell mask green, and Mito-green) and as-fabricated [Au_18_(TBBT)_12_(TFPP)_4_]^2+^ NC. Reproduced with permission from ref 131, Copyright 2025, Wiley. Confocal laser scanning microscopy images of live MCF-7 cells treated with (C) Au_18_SG_14_ and (D) Au_18_SG_12_MTPB_2_ for 1 h at 37 °C and costained with Lysotracker green and Mitotracker green, respectively. (E, F) The line profiles for Au_18_SG_14_, Au_18_SG_12_MTPB_2_. Reproduced with permission from ref 132, Copyright 2018, Royal Society of Chemistry.

Song et al. reported atomically precise [Au_18_(TBBT)_12_(TFPP)_4_]^2+^ NC (TBBT = 4-*tert*-butylphenthiophenol, TFPP = tri-(4-fluorophenyl) phosphine), which exhibited dual emission peaks (645 and 810 nm) in the aggregated state.^131^ To investigate their subcellular localization, colocalization experiments were performed on cells costained with the [Au_18_(TBBT)_12_(TFPP)_4_]^2+^ NC and commercially available subcellular-specific tracers. As illustrated in Figure 7B, the red fluorescence emitted by the NCs shows a high degree of overlap with the green fluorescence of the lysosomes. In contrast, minimal fluorescence overlap is observed between the NCs and either the cell membrane or mitochondrial green fluorescence. These findings suggest that the [Au_18_(TBBT)_12_(TFPP)_4_]^2+^ NC can be effectively internalized by lysosomes in living cells. These results indicate that MNCs have a higher propensity to be internalized by lysosomes within living cells. This perspective is further supported by the report from Zhu et al.^132^ They synthesized a small-molecule ligand containing both sulfhydryl and triphenyl phosphonium (TPP, a mitochondrial localization agent) structures, specifically identified as 4-mercaptobutyltriphenylphosphonium bromide (MTPB). The MTPB partially replaced -SG ligands on Au_18_SG_14_ to form Au_18_SG_12_MTPB_2_. After the two materials were incubated with Michigan Cancer Foundation-7 (MCF-7) cells, both of them demonstrated excellent cell uptake capabilities. Au_18_SG_14_ is mainly distributed in lysosome compartments of MCF-7 cells (Figure 7C, E), while Au_18_SG_12_MTPB_2_ mainly appeared inside the mitochondria (Figure 7D, F), which was further confirmed by costaining with Lysotracker green or Mitotracker green.^132^

Driven by the rapid advancement of cluster science, a growing number of NCs capable of emitting in the NIR-I and NIR-II regions have been successfully synthesized, fulfilling the requirement of high penetration in biological imaging.^133,134^ Cheng et al. demonstrated that Au_25_(SG)_18_ exhibited efficient binding affinity toward hydroxyapatite *in vitro*.^135^ Subsequent *in vivo* NIR-II imaging revealed that these Au_25_ NCs accumulated selectively in bone tissue, providing high contrast and a favorable signal-to-background ratio (SBR) (Figure 8A). When the skin and muscles are removed, nearly all bone structures can be visualized with high quality (Figure 8B), suggesting significant potential for application in guiding skeletal surgery.

**Figure 8:**
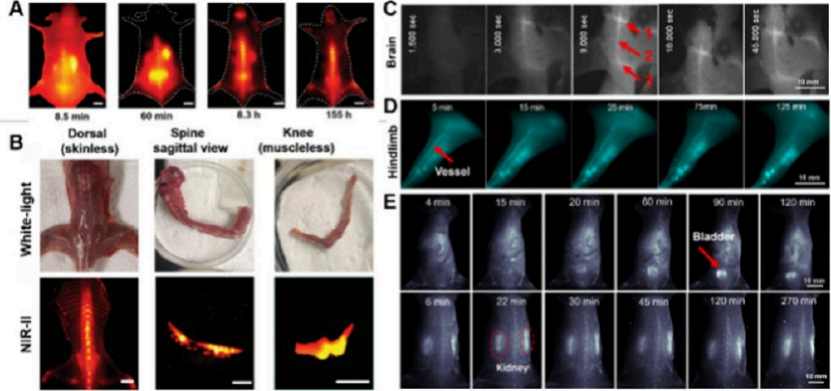
(A) Fluorescence imaging of C57BL/6 mice for the whole body in prone posture. (B) White-light and NIR-II fluorescence imaging of the above bones of ex vivo harvested at 12 h p.i. of Au NCs. Reproduced with permission from ref 135, Copyright 2020, Wiley-VCH. (C,D) NIR-II dynamic imaging of brain and leg vessels, respectively. The inferior cerebral vein, the superior sagittal sinus, and the transverse sinus are labeled 1, 2, and 3, respectively. (E) Real-time NIR-II signals of the bladder and kidney. Reproduced with permission from ref 136, Copyright 2023, American Chemical Society.

Zhang and his co-workers synthesized the Au_7_Cd_1_-MHA/MPA (MHA = 6-mercaptohexanoic acid), which showed NIR-II luminescence.^136^ The Au_7_Cd_1_-MHA/MPA NCs were intravenously injected via the tail veins of healthy mice to evaluate early stage cerebral perfusion and cerebral vascular status. At 3s postinjection (p.i.), the NCs were observed traversing from arterial vessels to venous vessels (Figure 8C). The NIR-II signals in small blood vessels diminished rapidly, and no significant signal was detected in most arteries and veins, except for the inferior vein, transverse sinus, and superior vein. Additionally, 5 min after injection, clear visualization of the leg vasculature was achieved and the signals remained stable for over 125 min (Figure 8D). Whole-body NIR-II imaging displayed that Au_7_Cd_1_-MHA/MPA could clearly delineate the abdominal blood vessels, spine, bladder, and kidneys, suggesting that its excretion primarily occurred through the urinary system (Figure 8E). These results indicate the promise of Au_7_Cd_1_-MHA/MPA for NIR-II dual-region imaging of the brain and vasculature.

Three-dimensional (3D) PL imaging extends the capabilities of conventional two-dimensional (2D) PL imaging by employing advanced technical methodologies to capture the spatial distribution of fluorescent signals from samples in 3D space.^137^ Notably, its ability to retrieve and reconstruct spatial dimensional information allows it to overcome key limitations inherent in 2D imaging, particularly regarding the spatial resolution, deep-tissue imaging capability, and high SBR.^138^ In recent years, the Zhang group has utilized MNCs to achieve 3D PL imaging for the diagnosis of different diseases.^139,140^

For example, they developed a NIR-II strategy for accurate breast cancer (BC) subtyping and 3D visualization using fluorescent Au_24_Pr_1_ NCs.^139^ Three different monoclonal antibodies targeting estrogen receptor (ER), progesterone receptor (PR), and human epidermal growth factor receptor 2 (HER2) construct the Au_24_Pr_1_ probes to identify BCs via NIR-II light-sheet microscopy (LSM). For the NIR-II 3D imaging of Luminal A, the fluorescence signals of ER and PR are clearly visible, while HER2 is insignificant, consistent with the expression patterns of the three biomarkers (Figure 9A-C). It can perform 3D imaging of biopsy samples with a wide field of view (∼500 μm) and at depths up to 500 μm without signal degradation. The x-y cross-sectional images of ER, PR, and HER2 show the SBR in representative regions. Background noise is substantially reduced, and compared to conventional NIR-I imaging, the SBR is enhanced by ∼3 times.

**Figure 9:**
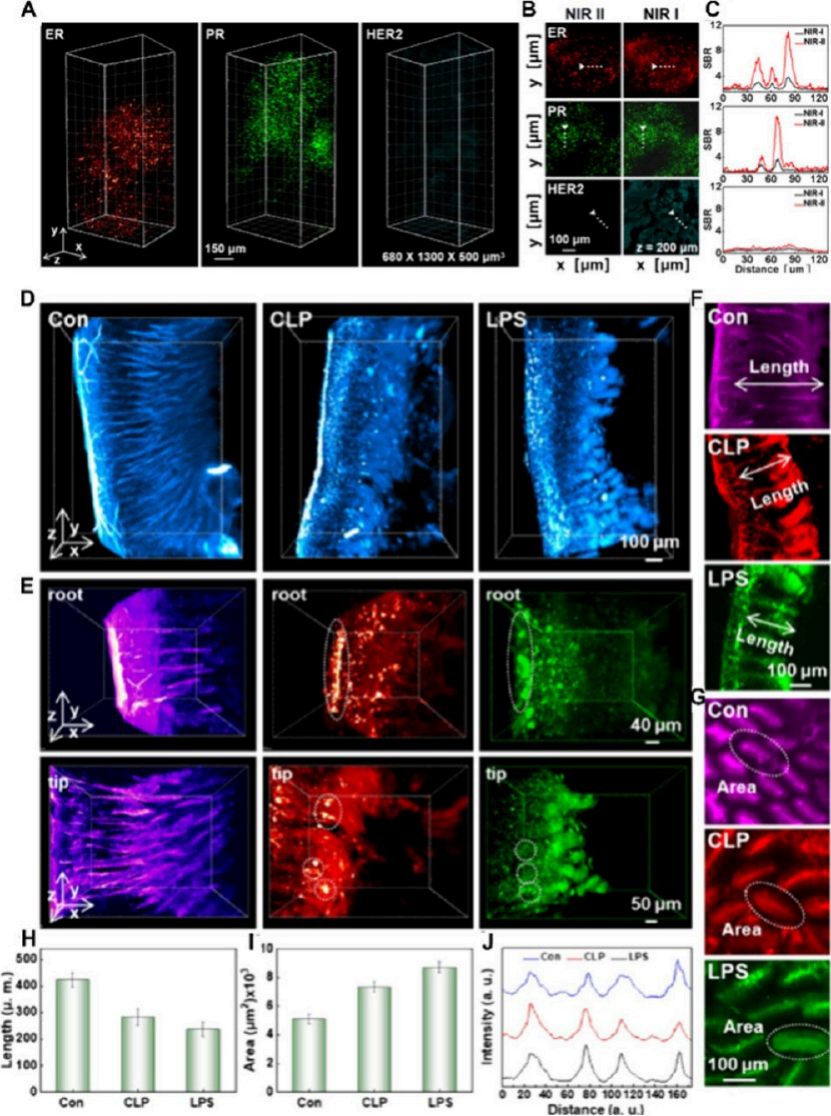
(A) 3D volumetric NIR-II LSM imaging of Luminal A biomarkers. (B) x-y cross-sectional images in (A) at 200 μm. (C) Normalized intensity profiles along the line shown in (B). Line intensity profiles are shown relative to the noise floor. Reproduced with permission from ref 139, Copyright 2024, American Chemical Society. (D) NIR-II imaging of the 3D structure of the mouse intestine. (E) Partially enlarged view of the root and tip of mouse intestinal villi. (F,G) Length and areas of the mouse intestinal villi of normal, CLP, and LPS, respectively. The quantitative analysis of the length (H) and the cross-sectional area (I) of intestinal villi. (J) Fluorescence intensities of intestinal cross sections were determined under different modeling methods. Reproduced with permission from ref 140, Copyright 2025, American Chemical Society.

In another study, Zhang et al. used Au_32_ NCs to detect multiorgan damage caused by sepsis in mice.^140^ The 3D visualization imaging of the mouse intestine was conducted using NIR-II LSM. As shown in Figure 9D,E, the villi structure of the small intestine in the control group was intact and regular. However, after cecal ligation and puncture (CLP) and lipopolysaccharide (LPS) damage, the morphology of the small intestinal villi became significantly shorter and blunter. Moreover, the integrity of the mucosal layer was severely damaged. It can be observed that the length of the small intestinal villi significantly shortened, while its surface area increased significantly (Figure 9F,G). The quantitative analysis gave results by measuring the length and area of the small intestinal villi (Figure 9H,I). The fluorescence signals of the intestinal cross-section were collected under different modeling conditions (Figure 9J). It can be observed that the signals of the intestinal villi in normal mice are regular and evenly distributed, while those of the model mice are disordered or even disappear.

### 3.2 CT Imaging

CT imaging is widely recognized as one of the most prevalent imaging modalities for disease diagnosis, attributed to its excellent deep tissue penetration, high spatial and density resolution.^141–143^ Iodinated small molecules are a type of commonly used CT contrast agent in clinical settings. However, owing to its rapid clearance by kidney, the imaging time is relatively brief.^144,145^ Furthermore, the administration of an iodine-based contrast agent may result in elevated osmotic pressure, potentially causing various types of tissue damage.^146,147^ As a consequence of these limitations, there is increasing interest focusing on the development of alternative CT imaging contrast agents.

With the development of nanotechnology, many NMs with good biosafety have been developed as contrast agents for CT imaging, such as metallic oxides,^148–150^ metal sulfides,^151–153^ and metal NPs.^154–157^ Among them, Au with a high-Z demonstrates a significantly higher X-ray absorption coefficient.^158–160^ In comparison to normal tissues, Au exhibits substantially higher radiation absorbency, which can be enhanced by up to 100 times within the keV energy range.^161^ Consequently, Au NCs composed of several or even hundreds of Au atoms can serve as highly effective contrast agents in CT imaging for the visualization of internal organs.

Basilion et al. utilized the highly specific biomarker (prostate-specific membrane antigen, PSMA-1) as ligands to synthesize Au_25_ NCs, achieving enhanced CT imaging and RT efficacy for prostate cancer, and rapid elimination by kidneys.^162^ As depicted in Figure 10A, the CT value of PSMA-positive PC3pip tumors 4 h p.i. was 374 Hounsfield units (HU), significantly higher than that of PSMA-negative PC3flu tumors (195 HU, 4 h p.i.). Furthermore, CT imaging of the mouse bladder clearly demonstrates the effective metabolism of Au_25_ NCs within 24 h (Figure 10B). Xie et al. reported GSH-stabilized Au_10–12_(SG)_10–12_ NCs that could be selectively deposited in tumor.^163^ After 6 h p.i., significant uptake at the tumor site (indicated by arrows) can be seen in both 3D and 2D CT imaging, and the corresponding CT value was determined to be 326 HU at the tumor site, which is significantly higher than that of normal tissue (207 HU) (Figure 10C). Gao and co-workers present BSA-protected Au NCs with robust X-ray attenuation.^164^ The synthesized Au NCs exhibited a slope of the HU value of 17.85, which is 4.3 times higher than that of the clinically used CT contrast agent (HU = 4.15), making them highly suitable for CT diagnosis of renal abnormalities (Figure 10D).

**Figure 10:**
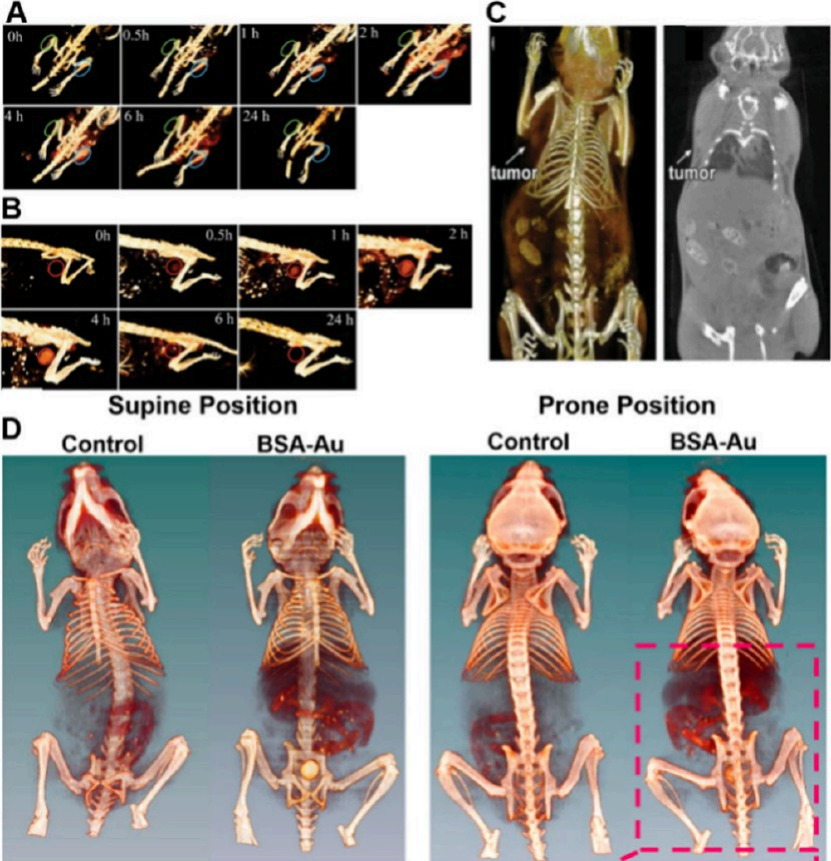
(A) *In vivo* 3D CT images of the PC3pip (right, blue) and PC3flu (left, green) tumor-bearing mice (indicated by blue and green ovals) before and at 0.5, 1, 2, 4, 6, and 24 h after intravenous injection of CY-PSMA-1-Au_25_ NCs (30 μg Au per g mouse). (B) *In vivo* 3D CT images of the bladders of mice (indicated by red circles) at each time point. Reproduced with permission from ref 162, Copyright 2019, Wiley-VCH. (C) Three (left) and 2D (right) small animal X-ray CT imaging of Au_10–12_(SG)_10–12_ at 6 h p.i. Reproduced with permission from ref 163, Copyright 2014, Wiley-Blackwell. (D) *In vivo* 3D CT images of saline and BSA-Au clusters-injected mice at 2 h post injection in the supine and prone positions. Reproduced with permission from ref 164, Copyright 2015, American Chemical Society.

### 3.3 PA Imaging

PA imaging is a 3D hybrid imaging technique that integrates optical and acoustic imaging modalities. By leveraging the high sensitivity of optical imaging and the deep penetration capabilities of acoustic imaging, it effectively compensates for the limitations of other imaging approaches.^165,166^ Due to their molecular-like properties and prominent multiabsorption bands in the visible to NIR region, MNCs exhibit significant potential for application in PA imaging when they have a high photothermal conversion.

Lin and colleagues synthesized Capt-stabilized Au_25_ NCs and further assembled these NCs into a mesoporous silica shell coating. Furthermore, the surface of the coating was functionalized with Nd^3+^-sensitized upconversion NPs (UCNPs).^167^ The Au_25_ NCs perform significant photothermal effects and PA imaging properties, and are further integrated with luminescence imaging of UCNPs to realize imaging-guided cancer therapy. As shown in Figure 11A, the PA intensity in tumor tissue progressively increases with the injection time, which can be utilized to facilitate visual guidance for tumor treatment. Zheng et al. reported the use of well-defined GSH-coated Au_25_ NCs (Au_25_(SG)_18_) for noninvasive monitoring of elimination by kidney through PA imaging.^168^ A single laser wavelength of 808 nm was utilized to excite Au_25_(SG)_18_ for reducing the interference of deoxyhemoglobin (Hb) and oxyhemoglobin (HbO_2_) (Figure 11B). The result demonstrated that the PA signal of Au_25_(SG)_18_ linearly correlates with the concentrations (Figure 11C). In addition, *in vivo* PA imaging was conducted, revealing that Au_25_(SG)_18_ was transported from the aorta to the renal parenchyma, then rapidly filtered into the renal pelvis for elimination with a temporal resolution of 1 s (Figure 11D,E).

**Figure 11:**
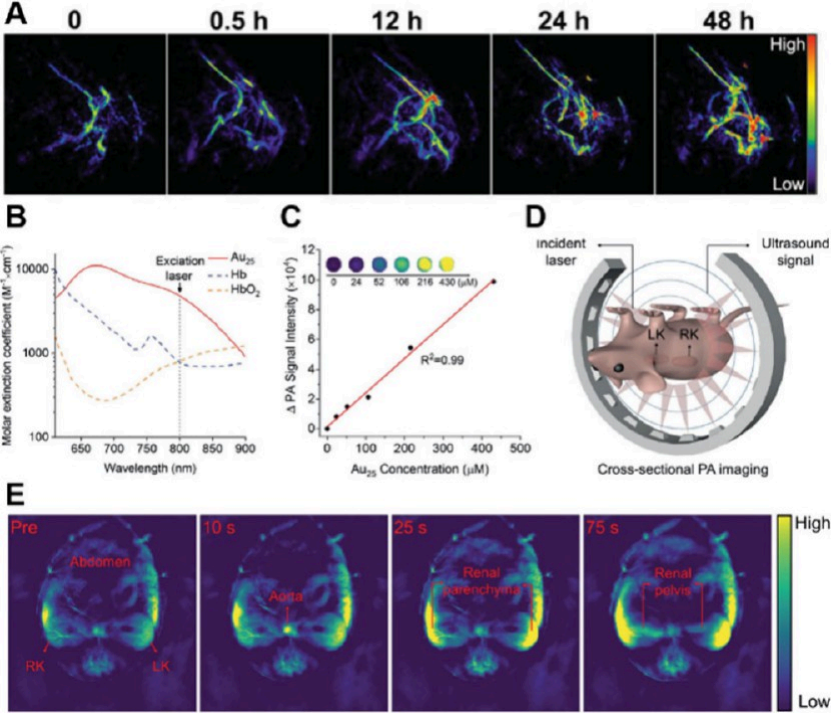
(A) *In vivo* PA images of tumor tissues after the intravenous injection of UCNPs@MS-Au_25_–PEG with different injection time. Reproduced with permission from ref 167, Copyright 2015, Wiley-VCH. (B) Molar extinction coefficient of Au_25_(SG)_18_ compared to that of Hb and HbO_2_ in the NIR range. (C) PA signal intensity increases as a function of Au_25_(SG)_18_ concentration measured by *in vitro* phantom studies. (D) Illustration of *in vivo* PA imaging experiment. (E) Representative preinjection PA images of normal mice and p.i. PA images of different time points. Reproduced with permission from ref 168, Copyright 2019, John Wiley and Sons Ltd.

### 3.4 Multimodal Imaging

The increasing demand for accurate medical diagnosis and treatment has spurred the development of various imaging technologies. However, a single imaging modality is not able to provide comprehensive diagnostic information. For example, CT imaging offers high spatial resolution and deep tissue penetration, but it exhibits limited sensitivity to soft tissues and often combined with magnetic resonance imaging (MRI).^169^ In order to overcome the limitations of a single imaging mode and further integrate the advantages of different imaging modes, a multimodal imaging nanoplatform combining two or more imaging methods can present the information on the entire organism more comprehensively.

Zhang et al. developed Au_15_ NCs functionalized with diethylenetriamine-pentaacetic acid dianhydride (DTPAA-Gd), which is an optimized multimodal imaging agent to enhance imaging ability.^170^ As shown in Figure 12A, the Au_15_NC- DTPAA-Gd displayed a great potential to be used as a multimodal imaging platform to achieve CT, near-infrared fluorescence (NIRF), and MRI imaging capability. Furthermore, the imaging components are mutually reinforcing, effectively overcoming the limitations caused by interference among imaging elements commonly observed in traditional multimodal platforms.

**Figure 12:**
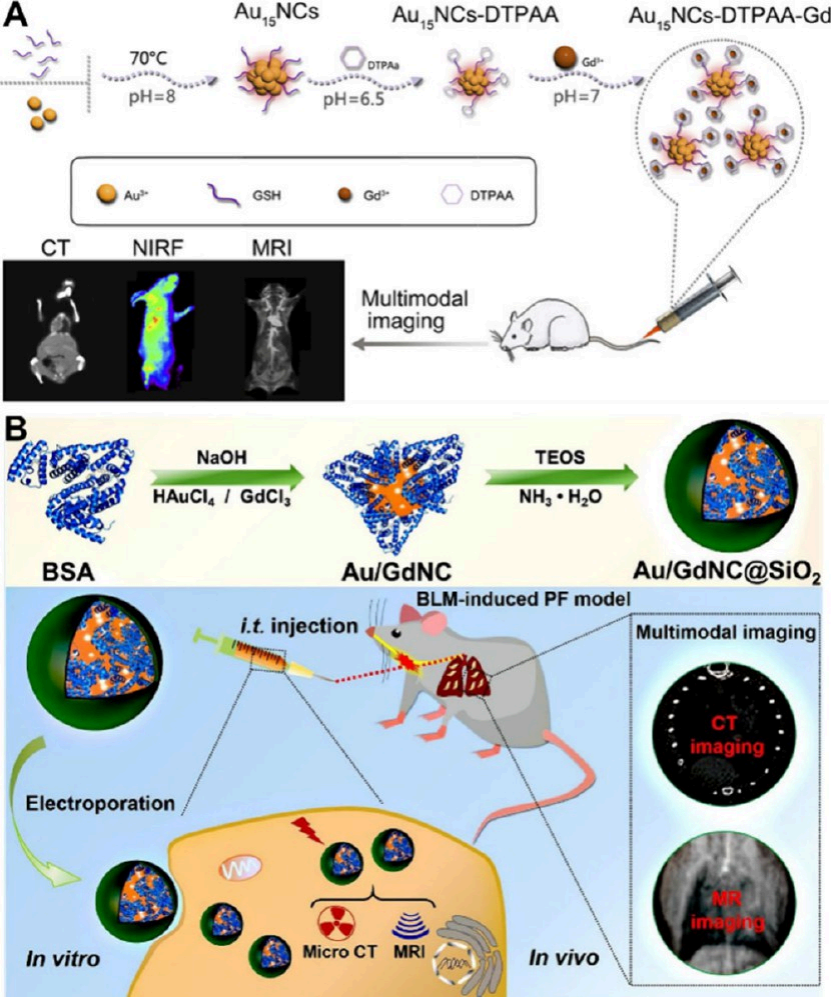
(A) Schematic illustration of the synthetic procedure and multimodal imaging for the Au_15_ NC-DTPAA-Gd nanohybrid. Reproduced with permission from ref 170, Copyright 2020, Dove Medical Press Ltd. (B) Schematic illustration of the synthesis of Au/GdNC@SiO_2_ nanotracer and its application for CT/MR dual-modal imaging tracking of the transplanted MSCs in a murine model of BLM-induced PF. Reproduced with permission from ref 171, Copyright 2020, American Chemical Society.

Zhang et al. reported BSA-stabilized bimetallic Au/Gd NCs that function as CT/MR dual-modal nanotracers (Figure 12B).^171^ A silica shell was overcoated onto the Au/GdNC surface (forming Au/GdNC@SiO_2_) to facilitate labeling and tracking of human mesenchymal stem cells (hMSCs) following transplantation into a bleomycin (BLM)-induced pulmonary fibrosis (PF) mouse model. No significant adverse effects were observed on the proliferation or differentiation capacity of the labeled stem cells. The integration of Au and Gd, both exhibiting strong X-ray absorption properties, into a single nanostructure generated a synergistic effect, thereby significantly enhancing both CT and MR imaging contrasts compared to those of either Au or Gd alone. In comparison with clinically used Ioversol and Magnevist, the developed nanotracers demonstrated a 42-fold increase in X-ray attenuation efficiency and a 6.5-fold improvement in MR *r1* relaxivity, respectively.

Since each imaging modality requires NMs with their corresponding functionalities, traditional multimodal imaging modalities typically necessitate the integration of various functionalities into a single nanoplatform. This approach not only involves a complex synthesis process but may also result in diminished retention of the original functionality. Therefore, for dual- or multimodal imaging applications, a single probe that incorporates multiple imaging contrast agents becomes a more favorable strategy. As previously mentioned, MNCs with attractive optical properties and remarkable X-ray attenuation properties provide specific advantages for this strategy, which are otherwise difficult for many large-sized NMs to achieve.

## 4 Disease Treatment

MNCs exhibit exceptional properties and have become promising candidates for bioimaging as well as demonstrating unique advantages in disease treatment. Many disease treatments have been developed based on MNCs. This section discusses the application of MNCs as nanomedicines in disease treatment and elucidates the key role they play in this field.

### 4.1 Photothermal Therapy

PTT is potential therapeutics in disease treatment due to its noninvasive and high selectivity.^172^ As typical physiotherapy, PTT utilizes photothermal agents (PTAs) to convert light energy into thermal energy, thereby increasing the temperature of surrounding tissues and inducing cell death.^173^ The multiple absorption bands of MNCs contribute to their excellent performance in photothermal conversion.

Recent research on NCs have focused on their photothermal conversion properties and has achieved some advancements.^174–178^ For example, Wu and his workers synthesized a Pd_8_(PPh)_2_(PPh_3_)_2_(S-Adm)_6_ NC with a single-atom-layered construction, which shows a high photothermal conversion efficiency (PTC, ∼73.3%) dissolved in toluene solution irradiated with a 808 nm laser at 1.0 W/cm^2^.^175^ And based on the systematic comparative experiments, the authors proposed that the electron transitions along the *x*-axis direction may primarily contribute to the induction of strong photothermal conversion. Fan and co-worker developed a Au25-based photothermal nanomachine, which responds to near-infrared photoexcitation and functions in mammalian cells and in vivo.^176^ Jin et al. reported Au_42_(SCH_2_Ph)_32_ with good photothermal conversion performance, which possessed a large temperature increase of ∼27 °C within 5 min (λ_ex_ = 808 nm, 1 W cm^–2^ at an ultralow concentration of 50 μg mL^–1^ in toluene (Figure 13A).^177^ Wan et al. synthesized all-alkynyl protected rod-shaped Au_9_(AgCu)_126_ NCs, showing a substantial temperature rise of ∼51.5 °C within 5 min (λ_ex_ = 660 nm, 0.5 W cm^–2^) and very high photothermal conversion efficiency of 84.7% at a concentration of 12 μM in N,N-dimethylformamide (Figure 13B).^178^ Sun et al. designed 155-nuclei silver NCs protected by thiacalix[4]­arene and cyclohexanethiol. When dispersed in chloroform at a concentration of 240 μM, the Ag_155_ NCs were able to achieve a temperature of 59.1 °C after 5 min of irradiation (λ_ex_ = 660 nm, 0.1 W cm^–2^) (Figure 13C).^179^ Wang et al. investigated the photothermal conversion capabilities of a series of bimetallic Au–Ag NCs. The results revealed that MNCs with weak fluorescence properties exhibited a notable advantage in terms of photothermal conversion (Figure 13D).^180^ Generally, the photothermal effect observed in atomically precise MNCs arises from the electronic transitions between distinct energy levels. Following photo absorption, the excitation energy is dissipated as heat during the internal conversion process from higher to the lowest excited state and the nonradiative electron–hole recombination process.^30^ In plasmonic metal nanoparticles, the excitation energy is first transferred to the electron gas, then to the metallic core’s phonons via electron–phonon coupling, and finally to the solvent via phonon–phonon coupling, generating the observed photothermal effect. The vibrational frequencies (∼10^12^ Hz) of MNCs are significantly higher than those of plasmonic metal NPs (∼10^10^ Hz).^30^ The photothermal efficiency of MNCs can largely surpass that of plasmonic nanoparticles.

**Figure 13:**
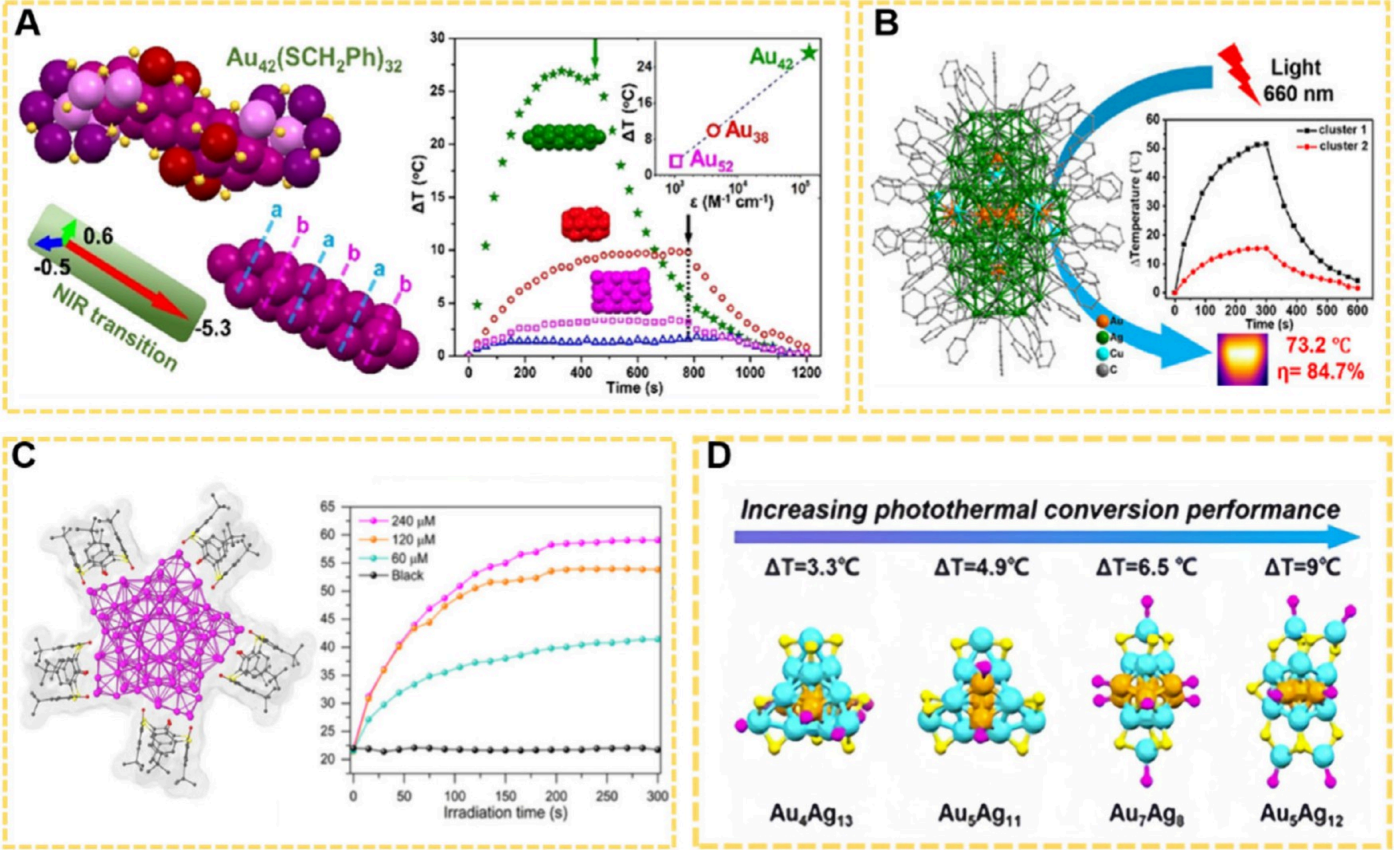
(A) Structure and temperature change curves of Au_42_(SCH_2_Ph)_32_. Reproduced with permission from ref 177, Copyright 2022, American Chemical Society. (B) Structure and photothermal conversion efficiency of Au_9_(AgCu)_126_. Reproduced with permission from ref 178, Copyright 2025, Wiley. (C) Structure and temperature changes of Ag_155_ NCs. Reproduced with permission from ref 179 Copyright 2022, John Wiley and Sons Ltd. (D) Structure and temperature change of different Au–Ag NCs. Reproduced with permission from ref 180, Copyright 2024, Royal Society of Chemistry.

The aforementioned studies demonstrated that MNCs, which possess favorable photothermal conversion properties, have the potential to serve as effective PTAs in PTT. In 2017, Katla and co-workers abandoned the traditional synthesis method of NCs and developed a breakthrough synthesis process for Au_25_ that does not require any complicated steps (even without stirring).^181^ The synthesized Au_25_(SG)_18_ NCs exhibited excellent performance on MDA-MB-231 breast cancer cells under 808 nm laser irradiation, which achieved 100% cell death, proving the application of PTT. In 2020, Jiang et al. reported that conjugation of indocyanine green (ICG) onto atomically precise GSH-coated Au_25_ (GS-Au_25_) NCs led to a molecular-like photothermal NPs (ICG_4_-GS-Au_25_).^182^ The combination significantly enhanced the ICG photostability and tumor targeting properties. Meanwhile, “off-target” ICG_4_-GS-Au_25_ could be eliminated from the body after being metabolized by the liver (Figure 14A). After 5 h p.i., the PTT of tumor was illuminated by a laser with a power density of 0.8 W/cm^2^ for 8 min. As shown in Figure 14B, the tumor temperature of mice injected with ICG_4_-GS-Au_25_ was greatly increased, while the ICG injected group is similar to that of PBS injected. The mice tumor volume injected with ICG_4_-GS-Au_25_ continued to shrink, and the tumor completely disappeared after 2 weeks (Figure 14C). As the research progresses, PTT is frequently employed in conjunction with other therapeutic modalities to enhance treatment outcomes,^183,184^ a topic that will be elaborated at section 5.

**Figure 14:**
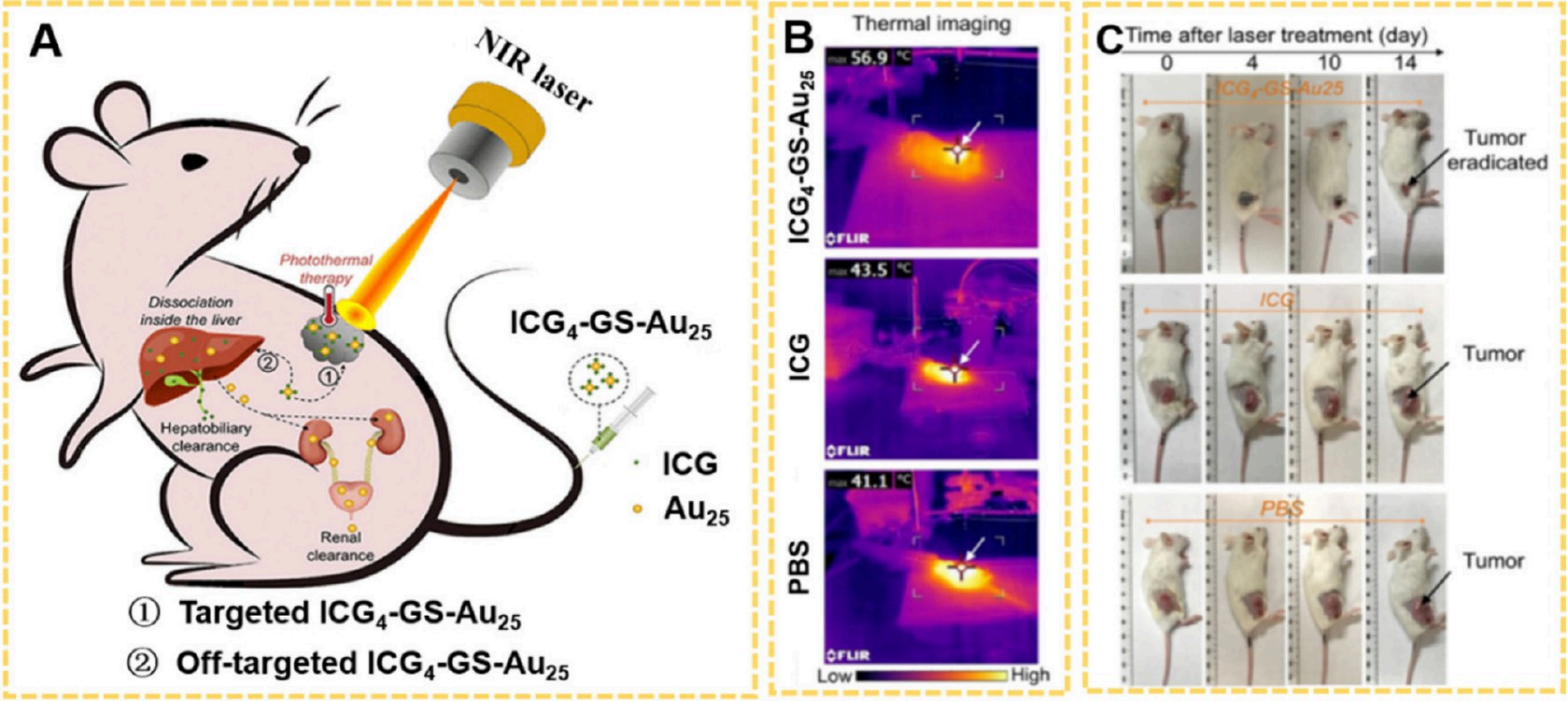
(A) Schematic illustration of the ICG_4_-GS-Au_25_ mediated cancer PTT and *in vivo* clearance pathways after dissociation in the liver. (B) Representative tumor thermal images of the mice receiving PTT treatment after 8 min of laser irradiation. White arrows indicate the tumors on mice. (C) Representative color images of the tumors on mice at different time points post PTT treatment. Reproduced with permission from ref 182, Copyright 2020, American Chemical Society.

### 4.2 Photodynamic Therapy

PDT is a therapeutic approach that employs photosensitizers (PSs) to produce substantial amounts of cytotoxic ROS upon laser irradiation, leading to the destruction of tumor cells or bacteria.^185^ This modality offers several advantages, including its noninvasive nature, minimal side effects, and absence of surgical complications.

Upon exposure to laser radiation, the electrons of the PSs undergo a transition from the ground singlet state (S_0_) to the higher-energy excited singlet state (S_1_). Since the excited-state electrons are inherently unstable, a portion of them undergo intersystem crossing and enter the lower-energy triplet state (T_1_). In comparison to the S_0_ states, excited-state molecules demonstrate enhanced oxidizing and reducing capabilities. Consequently, the electrons of PSs in T_1_ states can decay through two processes: Type I involves the generation of ROS, such as O_2_^•–^, hydrogen peroxide (H_2_O_2_), and hydroxyl radical (•OH), via electron transfer pathways; whereas Type II relies on the production of singlet oxygen (^1^O_2_) through energy transfer processes.^186,187^

When utilized as PSs in PDT, MNCs predominantly operate via a Type II mechanism. For instance, Liu et al. investigated the OXD-like activity of BSA-Au_25_ NCs and Au NPs under irradiation with different wavelengths.^188^ In comparison with Au NPs, BSA-Au_25_ generated a higher amount of ^1^O_2_ under visible light irradiation (Figure 15A). Zang et al. coassembled the Ag_29_ with the classic photosensitizer Ru­(bpy)_3_Cl_2_.^189^ Such an assembly exhibits a prominent enhancement of ^1^O_2_ under visible light irradiation compared to the case of Ag_29_ only (Figure 15B). Li et al. synthesized Au_38_S_2_(SAdm)_20_ NCs (SAdm = 1-adamantanethiolate) with a high production yield and display efficient ^1^O_2_ formation (Figure 15C).^190^ In addition, Galindo et al. synthesized TBA_2_[Mo_6_I_8_Ac_6_] NCs (TBA = tetra-*n*-butylammonium; Ac = acetate) and supported it on molecular organogels for the first time, which exhibited efficient generation of ^1^O_2_ upon illumination with white light (Figure 15D).^191^

**Figure 15:**
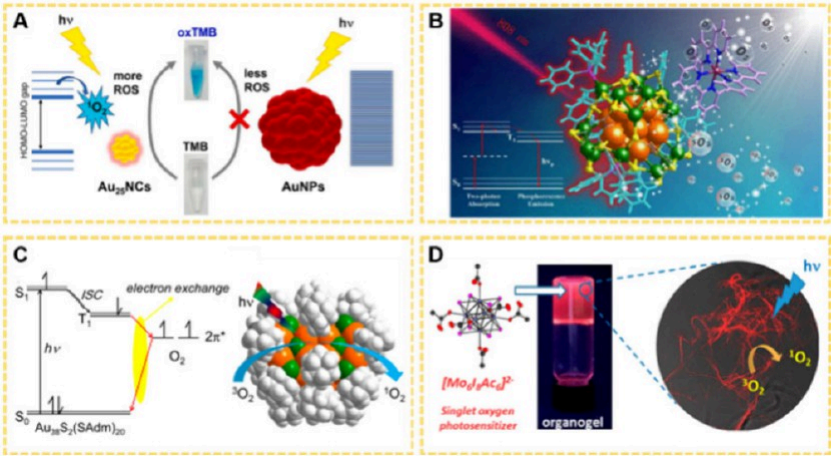
(A) Illustration of the mechanistic insight into the light-induced ^1^O_2_ production of Au_25_ NCs as compared to that of AuNPs. Reproduced with permission from ref 188, Copyright 2022, Elsevier. (B) Illustration of the mechanism for light-induced ^1^O_2_ production of Ag_29_–Ru­(bpy)_3_Cl_2_ assembly. Reproduced with permission from ref 189, Copyright 2022, American Chemical Society. (C) Mechanism of the Dexter-type electron exchange coupling between NCs and O_2_ for the generation of ^1^O_2_ (left) and illustration of the generation process on the Au_38_S_2_(SAdm)_20_ structure (right). Reproduced with permission from ref 190, Copyright 2017, American Chemical Society. (D) Schematic diagram of the generation of ^1^O_2_ by [Mo_6_I_8_Ac_6_]^2–^ dispersed organogel with laser irradiation. Reproduced with permission from ref 191, Copyright 2019, American Chemical Society.

Based on the photodynamic properties of MNCs, numerous studies of PDT have been reported. Yang et al. constructed a multifunctional dual-PS antitumor nanoplatform.^192^ By coating a layer of mesoporous graphitic carbon nitride (g-C_3_N_4_) on the core of the up-conversion NPs (UCNPs), and subsequently immobilizing ultrasmall Au_25_ NCs and polyethylene glycol, the UV–vis and strong NIR emissions of UCNPs can separately activate g-C_3_N_4_ and Au_25_ NCs to generate ROS, thereby achieving simultaneous activation of both PSs and improving the PDT efficiency mediated by a single NIR light excitation. The dual-PS system is more effective than a single-mode PDT effect (Figure 16A).^192^ Liu and his colleagues utilized Au_25_(Capt)_18_ NCs with photoreactive properties and further functionalized their surfaces by conjugating mitochondrial (Mito) targeting peptides, resulting in the formation of Mito-Au_25_.^193^ Subsequently, Mito-Au_25_ was adsorbed onto the surface of MnO_2_, forming a nanocomposite by electrostatic interaction. After being internalized by the cells, MnO_2_ is capable of depleting GSH. Subsequently, upon exhaustion of MnO_2_, the exposed Mito-Au_25_ selectively targets the mitochondria. Under 808 nm laser irradiation, it could enhance the PDT efficacy and increase the efficiency of ROS for killing tumor cells (Figure 16B).^193^

**Figure 16:**
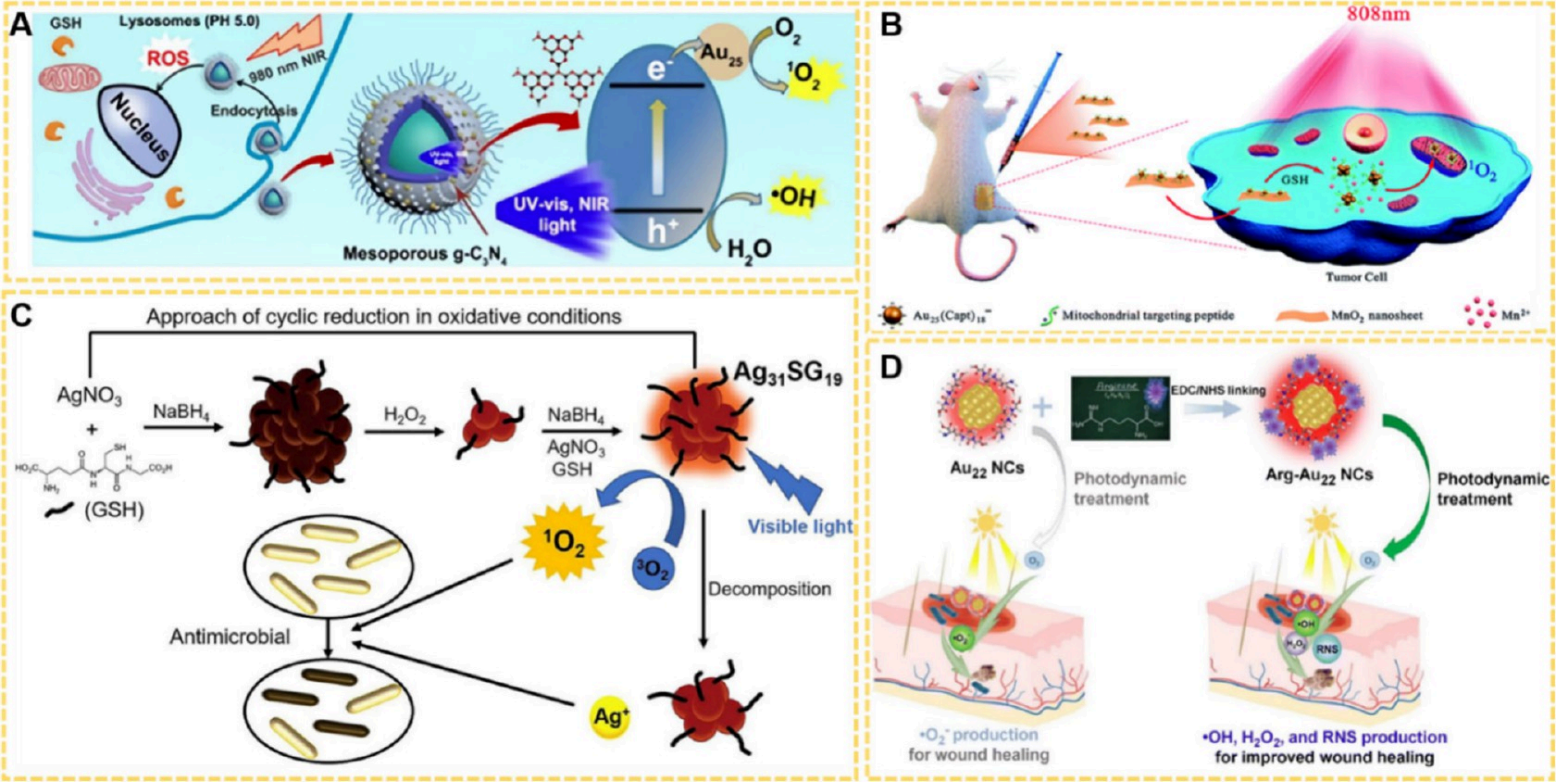
(A) Schematic representation of the double PSs system for the PDT treatment. Reproduced with permission from ref 192, Copyright 2017, American Chemical Society. (B) Schematic diagram of the assembling process and mechanism of Mito-Au_25_@MnO_2_ for *in vivo* PDT. Reproduced with permission from ref 193, Copyright 2021, Royal Society of Chemistry. (C) Schematic diagram of the synthesis process and antibacterial mechanism of Ag_31_SG_19_ NCs. Reproduced with permission from ref 194, Copyright 2024, Elsevier BV. (D) Schematic illustration showing the coupling of Arg with Au_22_ NCs for enhanced healing of bacterial-infected wounds. Reproduced with permission from ref 195, Copyright 2024, Royal Society of Chemistry.

Furthermore, MNCs exhibit remarkable efficacy in antibacterial activity using PDT. For example, Yuan et al. synthesized Ag_31_SG_19_ and investigated their ^1^O_2_ generation and antibacterial properties.^194^ The experimental results revealed that Ag_31_ NCs were capable of producing ^1^O_2_ under laser irradiation and releasing Ag^+^ ions, thereby exerting inhibitory effects against *E. coli* and *S. aureus* (Figure 16C).^194^ Sipaut et al. facilitated the healing of bacterially infected wounds by conjugating arginine (Arg) to the surface of Au_22_ NCs. The incorporation of Arg not only improved visible light absorption, enhanced PL intensity, and prolonged decay process of the NCs, but also altered the generation mechanism of ROS, while additionally enabling the production of reactive nitrogen species (RNS, e.g., NO). These improvements allowed the Arg-Au_22_ to synergistically combine the antibacterial effects mediated by ROS and RNS with the intrinsic antibacterial properties of NCs, exhibiting strong antibacterial activity (Figure 16D).^195^

### 4.3 Radiotherapy

Currently, RT is one of the most important clinical strategies for tumor treatment, primarily inducing the death of cancer cells through high-energy ionizing radiation.^196,197^ The introduction of ionizing irradiation can directly destroy cell nuclear material or indirectly produce large amounts of ROS.^198–200^ Sources of ionizing radiation can be categorized as exogenous beams, such as electrons, protons, and photons (X- or γ rays); or injection of radioactive isotopes to tumor sites through a minimally invasive process.^201,202^ MNCs, typically composed of high-Z metal atoms, exhibit significant potential for applications in RT.

In 2013, the Xie group pioneered the investigation of the role of precise Au_25_ NCs as radiosensitizer for RT in tumor treatment.^203^ In this study, two distinct ligand-protected Au_25_ (GSH and BSA) were synthesized to target the tumor with high specificity, leading to a substantial enhancement in RT efficacy. Tumor volume and weight exhibited significant reduction when GSH-Au_25_ NCs were utilized as radiosensitizers. In addition, the ultrasmall GSH-Au_25_ NCs (hydrodynamic diameter ≈ 2.4 nm) facilitated highly efficient renal clearance. By contrast, the larger hydrodynamic diameter of BSA-Au_25_ NCs (hydrodynamic diameter ≈ 6 nm) impaired renal clearance and induced hepatic injury. Subsequently, the same group reported GSH-protected Au_10–12_(SG)_10–12_ and Au_29–43_(SG)_27–37_ NCs, both of which exhibited exceptional performance as radiosensitizers in RT.^163,204^ These studies have exclusively focused on Au NCs stabilized by GSH, highlighting the significant potential of polypeptides as ligand-protected MNCs in cancer RT.^205,206^

When cancer cells are exposed to high-energy radiation therapy, the surrounding healthy tissues are at risk of incidental exposure to radiation.^207,208^ Consequently, the development of tumor-targeted sensitizers and the minimization of their distribution into healthy tissues can effectively mitigate radiation-induced damage to healthy tissues. Considering this issue, Zang et al. designed and synthesized a water-soluble Au_25_(S-TTP)_18_ (TPP-SNa = sodium 3-(triphenylphosphonio)­propane-1-thiolate bromide), which show both mitochondria-targeting ability and water-solubility.^209^ The Au_25_(S-TTP)_18_ both enhanced RT and triggered more serious immunogenic cell death (ICD), which promoted therapeutically effective antitumor immunity (Figure 17A). Additionally, the Zang group also reported levonorgestrel-protected Au_8_ and Pt_4_Au_4_ NCs, which generate a substantial amount of ROS to induce cancer cell apoptosis after irradiating with X-ray (Figure 17B,C).^208,210^

**Figure 17:**
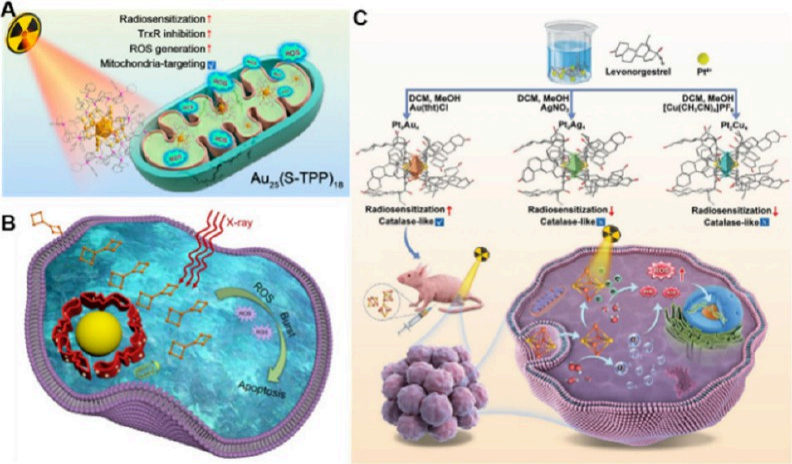
(A) Au_25_(S-TPP)_18_ used as radiosensitizer for RT in tumor treatment. Reproduced with permission from ref 209, Copyright 2023, American Chemical Society. (B) Au_8_ NCs for cancer RT via the ROS burst. Reproduced with permission from ref 210, Copyright 2019, American Chemical Society. (C) Schematic illustration of the synthesis of Pt_2_Au_4_, Pt_2_Ag_4_, and Pt_2_Cu_4_ clusters and the appplication of Pt_2_Au_4_ cluster in modulating tumor hypoxia to enhance RT efficacy. Reproduced with permission from ref 208, Copyright 2022, Wiley-Blackwell.

In the context of endogenous RT studies, Santos-Oliveira et al. synthesized radioactive gold NCs (^198^Au_25_(Capt)_18_) that demonstrated efficacy in eradicating prostate, breast, and melanoma cancer cell lines.^211^ The researchers further validated the safety of the radioactive NCs, confirming that they did not pose a risk of being pumped out of the cells by MRP1 (an ATP-binding cassette integral membrane transporter protein). These findings substantiate the potential of these NCs as nanomedicines for cancer treatment.

### 4.4 Nanodrug

During the course of disease treatment, NMs can directly serve as nanodrugs to treat various diseases, without requiring activation by external factors such as laser, ultrasound, or X-ray.^212^ MNCs are particularly well-suited for application as nanodrugs owing to their outstanding biocompatibility, renal clearance capacity, redox activity, antioxidant capabilities, enzyme-liking properties, and intrinsic antibacterial features.

MNCs with POD-like activity can directly catalyze the decomposition of excessive H_2_O_2_ in the tumor microenvironment, leading to the generation of ROS. The overproduction of ROS induces oxidative stress in tumor cells, which ultimately promotes apoptosis. This therapeutic approach is known as chemodynamic therapy (CDT) for cancer treatment.^213^ Li et al. successfully synthesized Cu_6_ NCs with a precisely defined structure.^214^ Under mildly acidic conditions (pH ∼ 6.0), the Cu_6_ structure was gradually destroyed, resulting in the explosive generation of ROS (Figure 18A). The Cu_6_ clusters have low cytotoxicity to normal cells but possess a concentration-dependent cytotoxic effect on tumor cells. These results confirm their biological safety, chemodynamic antitumor properties, and selective responsiveness to the tumor microenvironment.

**Figure 18:**
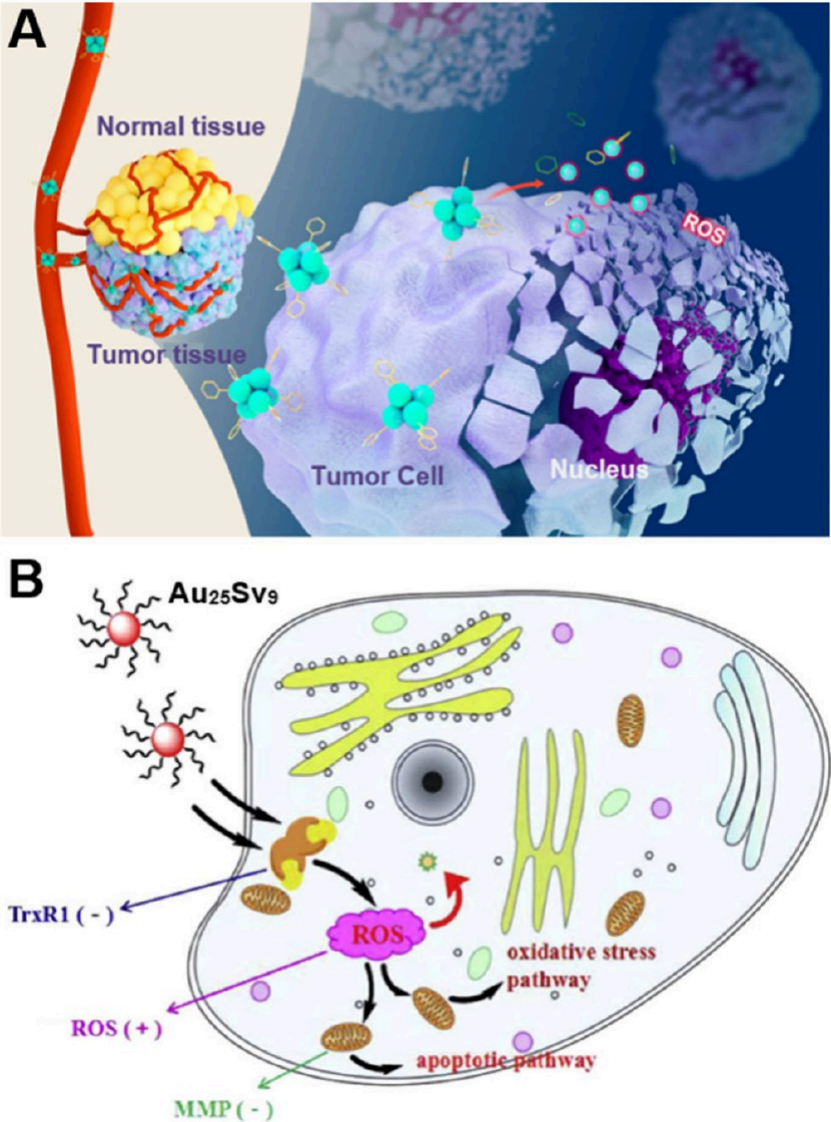
(A) Schematic of Cu_6_ NC for chemodynamic antitumor therapy. Reproduced with permission from ref 214, Copyright 2022, BioMed Central. (B) Schematic of the Au_25_Sv_9_ NCs against chronic lymphocytic leukemia. Reproduced with permission from ref 215, Copyright 2017, Science China Press.

Gao et al. synthesized Au_25_Sv_9_ NCs modified with a positively charged tripeptide (H_2_N-CCYG­GPKK­KRKPG-COOH, denoted as Sv), which can effectively penetrate the cell membrane of MEC-1 cells (human chronic lymphocytic leukemia cell line) and specifically bind to thioredoxin reductase 1 (TrxR1) in the cytoplasm.^215^ By increasing intracellular ROS levels, the mitochondrial membrane potential was disrupted, thereby activating the mitochondrial apoptosis pathway in MEC-1 cells. These findings suggest the potential therapeutic application of MNCs as therapeutic agents for chronic lymphocytic leukemia (Figure 18B).

Chronic oxidative stress in living organisms leads to the excessive production of ROS, which disrupt intracellular redox homeostasis and induce inflammation.^216,217^ Inflammation is a contributing factor in many diseases, including arthritis, neuroinflammation, and acute kidney injury, etc.^218–220^ Developing biosafety and efficient antioxidant NMs to combat excessive ROS is essential. Based on the enzyme-like catalytic properties, MNCs have the potential to alleviate diseases caused by oxidative stress.

Qian et al. employed Au_25_ NCs to modulate the transition of inflammatory M1 macrophages toward the anti-inflammatory M2 macrophages, thereby mitigating inflammation.^221^ Furthermore, Au_25_ effectively activates the ROS-mediated apoptotic signaling pathway by inhibiting thioredoxin reductase (TrxR), leading to a disruption in cellular redox homeostasis and induction of fibroblast-like synoviocyte (FLS) apoptosis (Figure 19A). In the adjuvant-induced arthritis rat model, Au_25_ significantly suppressed synovial hyperplasia and reduced inflammatory cell infiltration with minimal observed side effects. Lin et al. reported that atomic-level precise Au_22_(SG)_18_ NCs exhibited combined high CAT-like and SOD-like activities, thereby enabling efficient elimination of ROS.^222^ This capability can help restore the balance of the antioxidant system and achieve favorable therapeutic outcomes in high-fat-diet (HFD)-induced obesity treatment (Figure 19B). Zhang et al. engineered active Cu monomer sites within Au_22_ NCs through atomic-level manipulation.^223^ The resulting Au_21_Cu_1_ aggregate exhibited antioxidant activity that was 18 times greater than that of the undoped Au_22_ aggregate. Furthermore, its catalytic activity, comparable to that of hydrogen peroxidase, was enhanced by 90-fold, while its SOD-like activity increased by 3-fold. Notably, the Au_21_Cu_1_ aggregate effectively suppressed oxidative stress and inflammation in cisplatin-treated mouse models, particularly in the kidneys and brain (Figure 19C).

**Figure 19:**
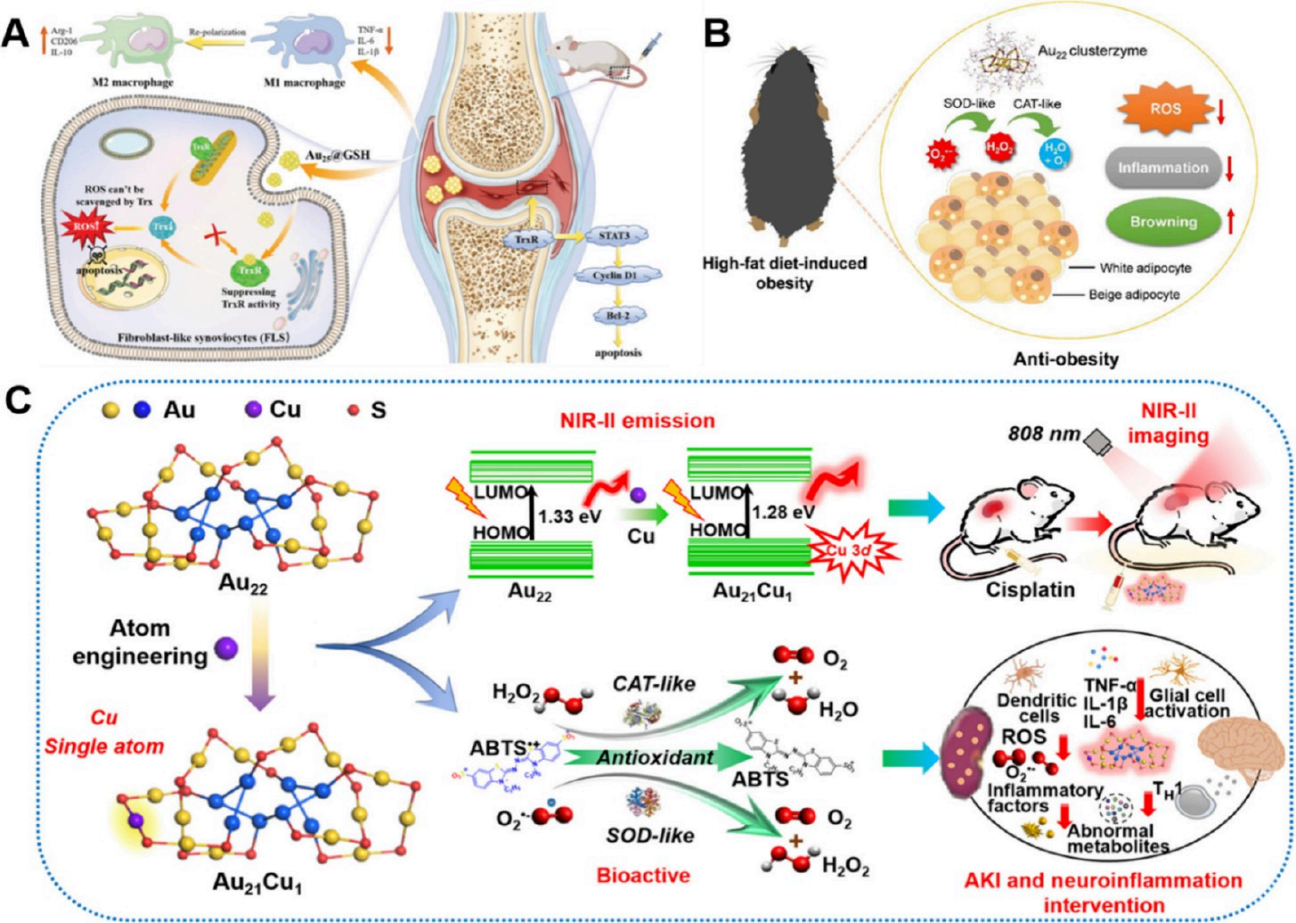
(A) Therapeutic mechanisms of Au_25_@GSH NCs in RA treatment with anti-inflammatory and antiproliferation combined therapy. Reproduced with permission from ref 221 Copyright 2022, Royal Society of Chemistry. (B) Schematic illustration for the treatment of HFD-induced obese mice with ROS-scavenging Au_22_ clusterzymes. Reproduced with permission from ref 222, Copyright 2024, Elsevier. (C) Schematic diagram of the properties and biomedical applications of Au_22_ clusters. Reproduced with permission from ref 223, Copyright 2023, American Association for the Advancement of Science.

### 4.5 Drug Delivery

Precision-targeted drug delivery is essential for improving drug utilization, reducing drug damage to normal tissues, and prolonging the continuous drug release at the lesion site.^224,225^ In order to address the precise drug delivery requirements, scientists have developed a variety of nanoscale drug delivery platforms, such as liposomes,^226–228^ nanovesicles,^229–231^ polymers,^232,233^ and inorganic NMs with porous structure.^234–236^ Among these materials, MNCs present a novel option as carriers for drug delivery due to their ultrasmall size, excellent biosafety and favorable properties.^237,238^ First, MNCs possess a large specific surface area, which enhances their drug-loading capacity. Drug molecules can be incorporated onto NCs through various methods such as physical adsorption, chemical bonding, and encapsulation within the inner layer.^239^ Second, the surface ligands of NCs containing multiple functional groups can be functionalized by introducing targeting groups. This modification facilitates precise targeting of lesion sites, thereby increasing the accumulation of drugs at the diseased location.^240–242^ Finally, the small size of MNCs facilitates the efficient penetration of the cell membrane, improving drug delivery efficacy. Moreover, the small size of NCs promotes rapid renal clearance reducing damage to normal tissues.^239^

Au NCs have been employed to enhance the intracellular delivery of antitumor drugs into neoplastic cells. Lin and his colleagues synthesized atomically precise Au_25_(MHA)_18_ NCs for the delivery of chemotherapeutic agent melittin (MEL) to human cervical cancer HeLa cells (Figure 20A).^243^ The NCs protected MEL from degradation and maintained prolonged cytotoxicity to HeLa cells. The antitumor activity exhibited by MEL can be attributed to the formation of pores on the cell membrane surface, a process that ultimately leads to cell lysis. This study pointed out that NCs can be used as delivery carriers for unstable peptides, improving the efficiency of chemotherapy. Xie et al. developed a traceable nanocarrier composed of Au_22_(SG)_18_ and chitosan, capable of combining with a chemotherapeutic platinum Pt­(IV) prodrug and the photodynamic aminolevulinic acid (ALA) via a bioconjugation method.^244^ This new nanocomposite (Pt­(IV)-ALA-Chito-Au_22_) exhibits pH-responsive drug release behavior. Compared to the individual components, Pt­(IV)-ALA-Chito-Au_22_ offers multiple advantages, including enhanced stability in physiological environments, improved PL, efficient cellular uptake without the need for targeting ligands, and favorable biocompatibility in normal cell lines (Figure 20B).

**Figure 20:**
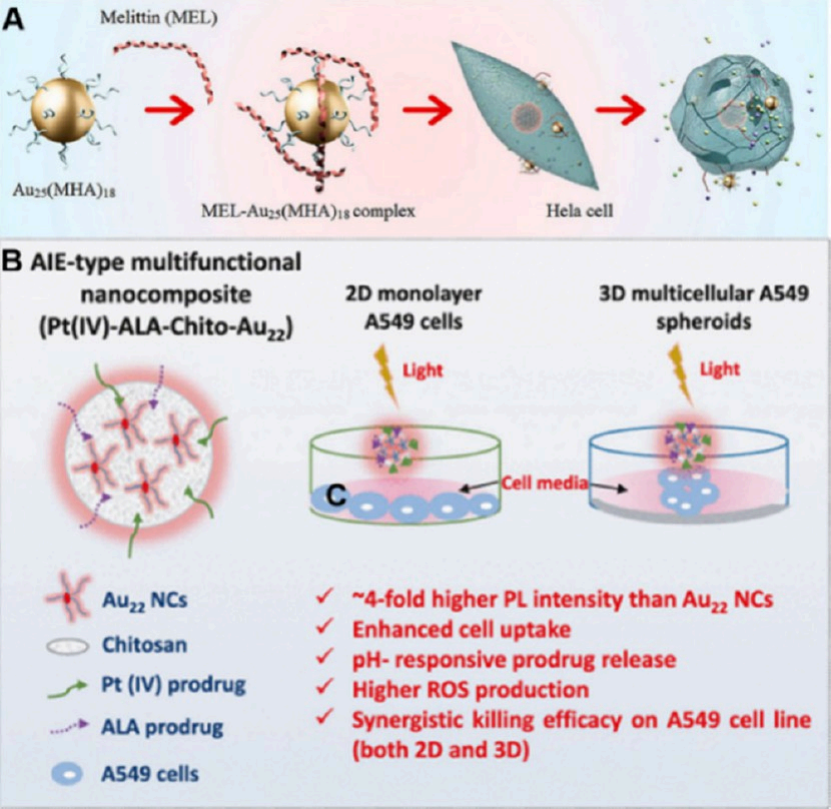
(A) Illustration of formation of MEL-Au_25_(MHA)_18_ complexes and delivery to HeLa cells. Reproduced with permission from ref 243, Copyright 2022, Editions de Sante. (B) Schematic illustration of the composition and intracellular action of Pt­(IV)-ALA-Chito-Au_22_. Reproduced with permission from ref 244, Copyright 2021, American Chemical Society.

The use of MNCs as drug delivery carriers has also been extended to the antibacterial field. Zhu et al. employed pentapeptide-protected Au NCs as drug delivery carriers for the antibiotic vancomycin (Van).^245^ The Van-loaded Au NCs demonstrated antimicrobial activity comparable to that of free Van against Gram-positive bacteria. Moreover, the release of Van was found to be proportional to the bacterial load, enabling demand-driven drug release. Additionally, the Au NCs exhibited strong luminescence, allowing the processes of drug loading and release to be monitored through fluorescence intensity changes.

## 5 Imaging-Guided Therapies

Noninvasive and precise diagnostic and therapeutic techniques represent optimal options for patients. Compared with conventional single-modality treatments, image-guided interventions that integrate diagnosis, monitoring, and therapy demonstrate notable advantages and are increasingly recognized as essential tools in clinical practice. These benefits stem from their ability to minimize physical trauma, reduce overall treatment costs, and provide real-time feedback, thereby enhancing the therapeutic efficacy. MNCs exhibit both imaging and therapeutic capabilities, making them suitable as integrated diagnostic and therapeutic probes.

Yuan et al. developed an ultrasensitive theranostic probe based on Au_44_MBA_26_ (MBA = 4-mercaptobenzoic acid) NCs, designed for NIR-II PL imaging-guided phototherapy and photoactivatable cancer immunotherapy.^246^ The probe is constructed by Au_44_MBA_26_ NCs with the immune checkpoint inhibitor 1-cyclohexyl-2-(5H-isodioxindolizine-5-yl)-ethanol (NLG919) via a ^1^O_2_-cleavable linker. The Au_44_MBA_26_-NLG probe enables deep-tissue NIR-II PL imaging for precise tumor localization, while simultaneously facilitating PTT and PDT with NIR photoirradiation, and controlled release of NLG919 for subsequent cancer immunotherapy (Figure 21A). Zhu et al. investigated the PTT/PDT properties of structurally precise oil-soluble Pt_1_Ag_28_ NCs, and dissolved them into the hydrophobic inner cavity of biodegradable amphiphilic chitosan derivatives (ACD) microcapsules through hydrophobic interactions.^247^ The positively charged Pt_1_Ag_28_@ACD with an appropriate particle size (∼60 nm) can effectively achieve passive targeted delivery, and can also be easily taken up by negatively charged cancer cells, thereby improving the targeting effect (Figure 21B). Moreover, enhanced PTT/PDT guided by aggregation-induced emission (AIE) fluorescence imaging was ultimately successfully implemented on the tumor.

**Figure 21:**
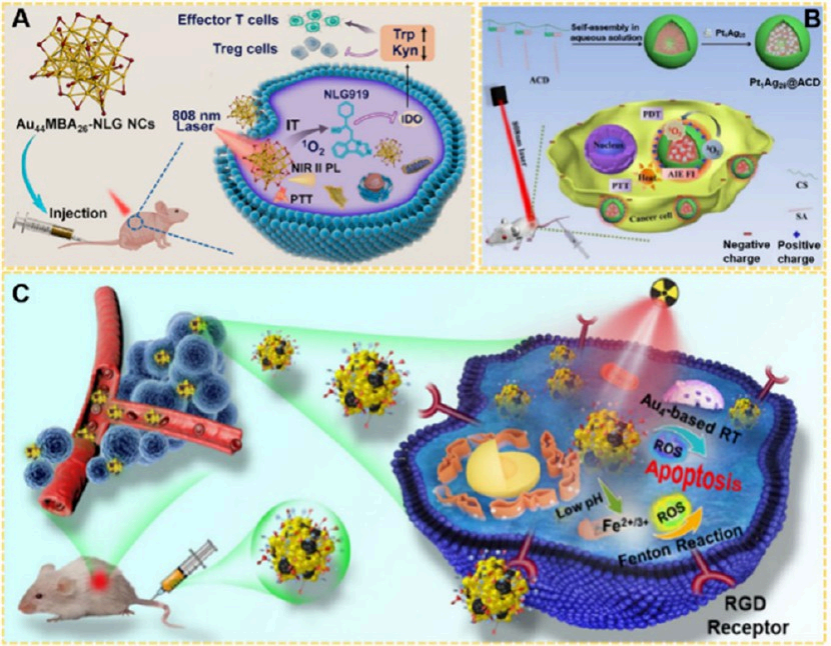
(A) Schematic diagram of the Au_44_MBA_26_-NLG probe used for imaging-guided tumor treatment. Reproduced with permission from ref 246, Copyright 2023, American Chemical Society. (B) Schematic illustration of Pt_1_Ag_28_@ACD platform for the self-assembly, enhanced targeted delivery and aggregation-induced fluorescence imaging assisted synergistic PDT/PTT effects. Reproduced with permission from ref 247, Copyright 2020, Elsevier. (C) Schematic illustration of the *in vivo* antitumor mechanism of Au_4_–IO NP-cRGD. Reproduced with permission from ref 248, Copyright 2021, BioMed Central.

Zang et al. assembled Au_4_ clusters with AIE property and iron oxide (IO) NPs in PBS buffer due to aurophilicity, and modified the surface with cyclic arginine-glycine-aspartic-phenylalanine-cysteine acid (cRGD) peptide (Figure 21C).^248^ The Au_4_–IO NP-cRGD demonstrates a favorable targeting efficacy against α_v_β_3_-integrin overexpressing tumor cells. Among the components, the Au_4_ cluster is capable of generating abundant ROS under X-ray irradiation, whereas the IO enhances the production of •OH through the Fenton-like reaction, thereby inducing synergistic damage to cancer cells. Additionally, the aggregated Au_4_ clusters and magnetic properties of the IO enable fluorescence imaging and MRI of tumors. This nanoplatform enables dual-mode imaging for the guidance of dual-modal combined tumor therapy.

Recently, the Song group prepared a novel Cu_*x*_Au_61‑*x*_ NC by doping Cu atoms into the Au_60_ NC.^110^ The incorporation of Cu atoms was found to significantly enhance both the NIR-II luminescence and enzyme-like activity, particularly under light irradiation of 808 nm, while maintaining an excellent photothermal conversion performance (Figure 22). To further evaluate its applicability, the Cu_*x*_Au_61‑*x*_ NCs were encapsulated with SiO_2_ to improve biocompatibility, followed by surface functionalization with folic acid (FA) to confer a tumor-targeting capability. It has been demonstrated that the penetration depth of Cu_*x*_Au_61‑*x*_@SiO_2_–FA was evaluated, in which the PL signal can be detected at a penetration depth of 9.00 mm and the imaging depth was determined to be ∼7.0 mm as the SBR decreased to ∼2. Adding to the excellent antitumor performance, the Cu_*x*_Au_61‑*x*_@SiO_2_–FA shows significant potential for application in NIR-II luminescence imaging-guided tumor phototherapy.

**Figure 22:**
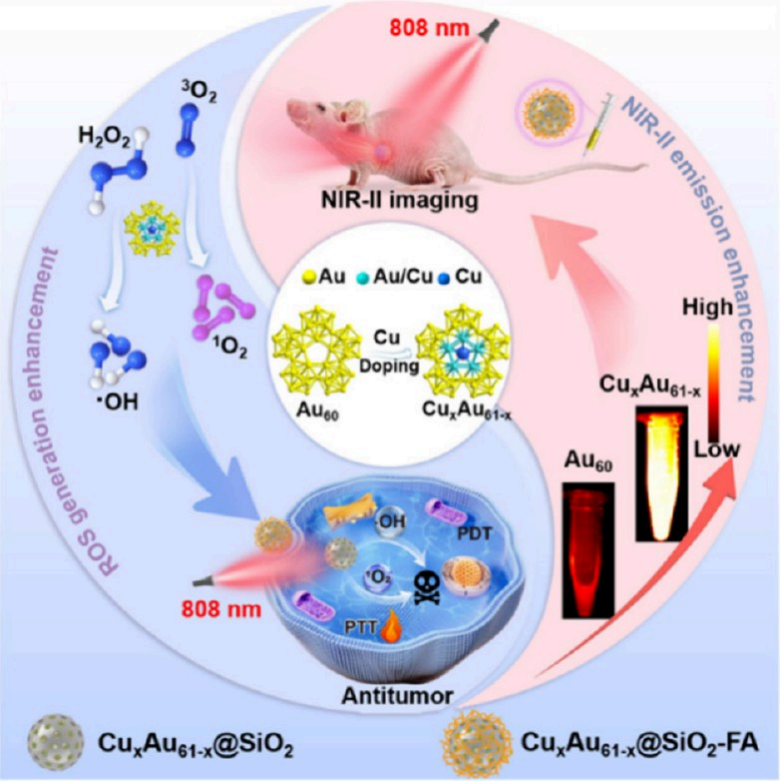
Schematic diagram of the synthesis and application of Cu_*x*_Au_61‑*x*_ NC in NIR-II luminescence imaging-guided tumor therapy.

## 6 Conclusion and Perspectives

In this review, we have provided an overview of the biologically relevant properties of atomically precise MNCs and their applications in biomedical fields, with the purpose of offering some categories of MNCs with specific functionalities (e.g., luminescence, photothermy, and enzyme-like activity) for biological applications. MNCs exhibit a multitude of advantages attributable to discrete electronic energy levels, which are a consequence of the quantum size effect. The primary properties of NCs that are pertinent to biological applications include their biosafety, optical absorption, PL, enzyme-like catalytic activity, and high X-ray absorption coefficient. For biomedical applications, we have summarized examples of NCs in biological imaging and disease treatment and emphasized the connections between applications and the related fundamental properties. Despite the noteworthy advancements in the utilization of atomically precise MNCs for biological applications, some challenges still exist and, thus, call for future developments.

(1) Although a series of well-defined MNCs, including mono-, bi- and polymetallic NCs, have been reported, the majority of these NCs are stabilized by hydrophobic ligands, whereas those protected by hydrophilic ligands remain relatively scarce. Having good dispersion and stability of NMs in aqueous solution are prerequisites for their applications in the biomedical field. Therefore, developing a library of water-soluble MNCs with atomic precision and high stability is still necessary. The current synthesis methods for hydrophilic MNCs are relatively limited, and the products tend to be polydisperse, which requires complicated separation/purification processes and thus results in inefficient outcomes. For future work, it is imperative to devise new synthetic strategies for the preparation of hydrophilic MNCs in high yield and with monodispersity by controlling the reaction conditions or by selectively exploring a range of synthetic strategies.
(2) In parallel to the development of aqueous-phase MNCs, it is highly meaningful to develop strategies of utilizing the organic soluble MNCs in biomedical applications. In the past decade, the synthesis of oil-soluble MNCs received significant attention for the purpose of crystallization in organic solution. Through systematic optimization of diverse synthetic strategies, a rich library of structurally precise and functionally advanced MNCs have been successfully developed. If these oil-soluble MNCs can be encapsulated into the internal cavities of hydrophilic biomaterials, such as hydrophilic mesoporous materials, small-molecule micelles, etc., to improve their biocompatibility, the efficiency of their biomedical application would be greatly improved.^249^ Notably, Liu et al. recently developed a general strategy for simple and efficient transfer of oil-soluble NCs into the aqueous phase, in which an amphiphilic polymer (Pluronic F127) was used to wrap the NCs.^250^ More importantly, this approach preserves the ultrasmall size (∼2 nm) of the MNCs, which is critical for enhancing their biological safety and functionality.
(3) Atomic-level MNCs have been demonstrated to possess not only biocompatible but also renal clearable. These NCs do not induce substantial physiological toxicity in organisms, even at elevated doses. However, it also significantly reduces the circulation time of MNCs in the bloodstream *in vivo* and decreases their selectivity and retention time at the lesion site, which can have a substantial impact on the therapeutic efficacy of MNCs on lesion. Consequently, the development of certain targeting groups (e.g., FA, peptides, antibodies) to modify the MNCs is needed in order to enhance their targeting specificity and retention time in the diseased tissues,^251^ while off-targeted MNCs can be rapidly metabolized.
(4) Although organic soluble MNCs with much higher QYs have recently be obtained, and several strategies have been reported to improve the luminescence QY of the NCs, such as ligand engineering, heterometal doping, aggregation, etc. the PLQY of water-soluble MNCs remain relatively low. Compared to commercial fluorescent dyes, a considerable performance gap still exists in the MNCs research, which poses a major challenge for their clinical application such as fluorescence imaging-guided surgery. Therefore, the development of water-soluble MNCs with significantly improved PLQY toward the NIR region (particularly 900–1700 nm) is urgently needed in order to enable a wider application of MNCs as PL probes in clinic.
(5) Last but not least, in addition to the afore-discussed functionalities of MNCs, magnetism has also been reported in some MNCs, such as the charge-neutral Au_25_(SR)_18_, but there have been no biomedical applications yet. Such magnetic MNCs may be promising as a new class of MRI probes and for other applications such as the probing of brain’s weak magnetic fields and neuron activity. Another functionality pertains to the chirality of MNCs, which may hold promise in chiral sensing of biomolecules such as amino acids and biocatalysis. Future work may explore these functionalities of MNCs for their potential in biomedical applications.
  Overall, the new class materials of atomically precise MNCs are unique in that they can be made with precise composition and structure at the atomic level. Biological functionalization of MNCs endows them with targeting capability and definitive valency (e.g., monovalency for monofunctionalized probes^249^ and similarly, polyvalent probes^252^). The new generation of MNC-based probes may provide unprecedented opportunities in bioimaging and therapy, as well as mapping the binding sites on proteins for drug discovery. The mechanistic understanding from the NMC-based biological probes will accelerate the rational design of theranostic strategies and contribute to the biomedical development. We expect that atomically precise MNCs will find greater opportunities in future efforts of expanding their applications in biomedicine.
